# Restorative museum environments: emotional coping strategies for people living with chronic multimorbidity

**DOI:** 10.3389/fpsyt.2025.1677909

**Published:** 2025-10-23

**Authors:** Keren Mao, Sijin Qian

**Affiliations:** ^1^ School of Art & Design, Zhejiang Sci-Tech University, Hangzhou, China; ^2^ College of Fine Arts and Design, Hebei Normal University, Shijiazhuang, China

**Keywords:** chronic disease co-morbidity, museum experience, healing environment theory, emotion regulation, health and well-being

## Abstract

**Objective:**

With the growing population of individuals suffering from chronic disease comorbidities, mood disorders have emerged as a critical factor adversely impacting their quality of life. As a potential form of restorative environmental intervention, museum spaces possess unique advantages in fostering emotional recovery and providing mental health support. This study aims to explore museum design strategies grounded in restorative environmental therapy to enhance emotional regulation experiences for patients with chronic comorbidities.

**Methods:**

This research integrates the Kano model, Quality Function Deployment (QFD), and the Pugh Matrix (Platts’ Matrix) to systematically identify, classify, and prioritise the emotional regulation needs of patients with chronic comorbidities within the context of museum environments. By establishing a mapping relationship between the characteristics of healing environments and specific spatial design elements, the study develops a structured design framework tailored to emotionally supportive museum spaces.

**Results:**

Findings indicate that museum designs optimised through principles of healing environment therapy significantly enhance emotional recovery in patients with chronic comorbidities. Improvements were observed in aspects such as spatial layout, sensory stimulation, user interaction, and perceived sense of belonging. Compared to conventional museum spaces, the optimised designs yielded higher emotional regulation scores and markedly reduced indicators of anxiety, loneliness, and mood instability.

**Conclusion:**

This study confirms the efficacy of healing environment-based design strategies in museum settings for regulating the emotional states of patients with chronic comorbidities. It proposes actionable design interventions and strategic pathways that offer both theoretical foundations and practical guidance for the future development of health-promoting public spaces. Moreover, it broadens the application of restorative environmental therapy within the cultural sector.

## 1 Introduction

In 2008, the World Health Organization (WHO) formally introduced the concept of multiple chronic diseases, or comorbidities, defined as “the co-existence of two or more chronic diseases in the same individual with a chronic condition.” This definition emphasizes a person-centered perspective that focuses on the individual experiencing multiple conditions (1). Similarly, the U.S. Department of Health and Human Services defines Multiple Chronic Conditions (MCC) as the presence of two or more chronic illnesses within a single patient (2).

Recent data indicate that China has the largest elderly population in the world aged 60 years and older. Among this demographic, approximately 180 million individuals—accounting for 75% of the elderly population—suffer from chronic illnesses, with the prevalence of multiple chronic conditions (MCC) reaching as high as 65.14% (3). MCC significantly impairs the quality of life of older adults and is associated with heightened psychological distress. Psychological distress refers to non-specific negative psychological states, such as anxiety and depression, often triggered by high-stress circumstances (4). Research has shown that elderly patients with MCC encounter various psychosocial stressors, including fear of disease progression, difficulties in self-management, and substantial financial burdens. These factors increase the risk of developing anxiety and depression, thereby adversely affecting treatment adherence and recovery outcomes (5). At present, there are no specific pharmacological treatments for psychological distress induced by MCC. Conventional drug-based therapies primarily target isolated symptoms and have not achieved satisfactory holistic outcomes. Consequently, non-pharmacological interventions have emerged as a crucial component in managing the psychological well-being of patients with MCC (6).

Currently, a range of non-pharmacological interventions for managing multiple chronic conditions (MCC) has emerged, including Cognitive Behavioral Therapy (CBT) (7), Mindfulness-Based Stress Reduction (MBSR) (8), Music Therapy (9), Exercise Therapy (10), and Restorative Environment Therapy (RET) (11).

Each therapy offers distinct focal points, yet they share common therapeutic benefits for older adults with chronic comorbidities. These benefits include reductions in psychological stress, enhancements in psychological resilience and emotional stability, heightened perceptions of social support, and improvements in overall well-being. Collectively, they have demonstrated efficacy in alleviating anxiety and depression, supporting patients in coping more positively with the challenges of chronic illness while maintaining emotional balance (12). Furthermore, by fostering emotional and social connectedness, these interventions help reduce feelings of loneliness and strengthen a sense of belonging, thereby enabling patients to attain inner peace and fulfilment. As a result, these approaches significantly enhance patients’ quality of life (13).However, existing treatments and design approaches predominantly focus on single modalities and relatively simple experiences, failing to address the multidimensional needs of MCC patients and thus falling short of meeting their complex requirements. This study innovatively proposes leveraging museum environment design as a means to support emotional regulation among MCC patients, with the ultimate aim of improving their quality of life and psychological well-being.

To achieve this goal, we adopt a set of systematic analytical tools, including the Kano model, Quality Function Deployment (QFD), and the Pugh matrix. The Kano model is employed to identify the emotional regulation needs of MCC patients and to examine their satisfaction with specific design attributes of museum environments. QFD is then applied to translate these needs into concrete functional requirements for museum design. Finally, the Pugh matrix is used to evaluate and compare alternative design solutions, thereby selecting the most suitable museum environment design for MCC patients.

The overarching objective of this study is to explore in depth how museum environment design can facilitate emotional regulation in MCC patients and to propose corresponding design strategies. It is expected that the findings will provide both theoretical insights and practical guidance for future museum design and care services targeting the elderly population.

## 2 Museum environment and emotional regulation

### 2.1 Museum-based care services for patients with MCC

Museums function as both symbols and custodians of human civilization, entrusted with the responsibility of connecting art to the public. When museums curate and present content that responds to public needs, they become cultural snapshots of their era (14). As cultural spaces, museums also provide a setting for art therapy, whose professional aims align closely with the educational missions of museums (15). According to the 2022 definition published by the International Council of Museums (ICOM), “education is the whole purpose of all museum activities,” reaffirming that public education has long been central to the museum’s existence (16). Moreover, the role of museum education has evolved beyond the transmission of information about artworks to emphasize the personal engagement and meaningful experiences that visitors derive from their interactions with museum content (17).

With the growing emphasis on the public service functions of museums, museum visits are increasingly being recognized as a form of “social prescription” aimed at enhancing the emotional well-being of vulnerable populations (18). In recent years, numerous museums worldwide have begun to explore their therapeutic potential. For example, the Museum of Modern Art (MoMA) in New York has collaborated with arts and cultural organizations such as CultuRunners and the World Health Organization’s Art and Health Programme to launch the Healing Arts initiative. MoMA has also introduced other programs focused on emotional wellness, including Artful Practices for Well-Being (2020) and The Healing Power of Art (2021) (19). Similarly, the Montreal Museum of Fine Arts (MMFA) has partnered with healthcare institutions to provide art-based interventions aimed at improving patients’ mental health. Through guided art appreciation and participatory experiences, these programs seek to alleviate symptoms of anxiety and depression, while enhancing overall quality of life (20).

As museums increasingly engage in care practices for older adults with special needs, theoretical literature and academic research on museum-based art therapy have correspondingly expanded. In their study Art Therapy in Art Museums: Promoting Social Connectedness and Psychological Well-Being of Older Adults, Bennington et al. (2016) demonstrate that organizing art viewing sessions and creative activities within museum environments enables older adults to express emotions, reflect on personal memories, enhance social interaction, and improve psychological well-being (21). Similarly, Morse et al. (2023), in their pilot mixed-methods study titled Exploring the Potential of Creative Museum-Led Activities to Support Stroke In-Patient Rehabilitation and Well-Being, found that creative, museum-led interventions positively impact stroke inpatients’ emotional states and facilitate better psychological adjustment to post-stroke challenges (22).

### 2.2 Exploring restorative environmental therapy through the lens of flow theory

At the end of the twentieth century, psychologist Mihaly Csikszentmihalyi introduced Flow Theory to describe a distinct psychological state characterized by deep immersion and optimal engagement in an activity (23). He argued that the flow state involves intense focus, intrinsic enjoyment, and a diminished awareness of time and surroundings, allowing individuals to become fully absorbed in the present moment. Csikszentmihalyi’s research indicates that flow typically emerges when individuals engage in tasks that are both challenging and manageable, prompting them to perform at their highest capability and experience profound psychological satisfaction. Flow Theory has since demonstrated significant relevance in the domain of restorative environmental therapies, offering a foundational theoretical framework for understanding psychological healing processes (24).

Restorative Environment Therapy (RET) seeks to alleviate stress, rejuvenate mental well-being, and enhance overall health by engaging individuals in natural or purposefully designed therapeutic settings (25). The conceptual foundation of RET can be traced to Frederick Law Olmsted, the influential American landscape architect, who intentionally integrated natural environments into his designs after observing their ability to relieve physical fatigue and psychological distress (26). Later research in environmental psychology has further validated the restorative effects of ecological settings, demonstrating their role in facilitating psychological recovery, mitigating disease progression, supporting emotional regulation, and fostering self-actualization. In particular, remote natural environments—such as forests, wilderness, and seashores—have shown remarkable therapeutic benefits when compared to urban settings.

Building on this foundation, Rachel and Stephen Kaplan proposed a cognitive framework for understanding the unique attributes of restorative environments. They identified four core components: Being Away, Extent, Fascination, and Compatibility, each of which contributes to attentional recovery and emotional restoration (27). These components are summarized in **Table 1** (28–30), which outlines their psychological functions and their relevance to healing-oriented design. When present in a given environment, these features support the emergence of immersive mental states similar to psychological “flow”—states characterized by deep engagement, focused attention, and intrinsic reward. Such experiences not only reduce stress but also foster long-term feelings of pleasure, fulfillment, and both psychological and physical restoration.

**Table 1:** Characteristics, requirements, and implementation strategies of restorative environments.

Characterisation	Request	Implementation method
Being Away	①Leaving the regular living environment. ②Avoid things that require the use of directed attention.	①Change of environment, e.g. seaside, mountains, rivers and lakes, forests, pastures, etc. ②Psychological adjustments, such as changing the content of thinking, sitting in meditation, etc.
Extent	Have a rich content and rational structure that attracts and occupies the individual's vision and mind, prompting him or her to fully engage in a deeper exploration and perception of the environment in which he or she lives.	Doesn't necessarily need to be large in physical size, as long as the content and structure are adequate.
Fascination	Information in the environment gains an individual's attention without effort.	Utilising the tranquil qualities of the natural environment to induce a moderate level of spontaneous attention in individuals. Achieving soft attraction.
Compatibility	①The environment is tailored to the individual's preferences and goals. ②Individual decision-making is adapted to the needs of the environment, and the process is a two-way interaction.	Positioning the target group and selecting the right environmental elements to provide an effective and accurate healing environment.

Within the framework of mindstream theory, restorative environmental therapies encourage individuals to activate their inner potential by engaging both their bodies and senses in the pursuit of a “peak moment.” Such experiences not only enhance self-efficacy but also deepen one’s sense of connectedness to the surrounding environment. Similar to mindfulness practices, which require individuals to strike a balance between challenge and skill, restorative environments offer calibrated sensory stimuli and cognitive engagement that facilitate deep psychological healing. These environments help reduce emotional burden and promote sustained well-being. Empirical studies have shown that restorative environmental interventions are effective in alleviating anxiety, depressive symptoms, and psychological exhaustion among individuals with multiple chronic conditions. By fostering more adaptive responses to life stressors, these therapies contribute to an enhanced overall sense of well-being (31).

### 2.3 Research problem

The demand for emotion regulation services for individuals with multiple chronic conditions (MCC) is steadily increasing in response to the global ageing trend and the rising prevalence of chronic diseases (32). In an effort to enhance the quality of life for this population, museums have begun to explore their potential role in providing emotionally supportive services. However, current research and design practices often emphasize simplistic experiences and unimodal interventions, with limited attention to the complex and multidimensional emotional needs of MCC patients. This has resulted in widespread design homogenization, which significantly undermines the effectiveness of these interventions in addressing the cognitive and affective challenges faced by this group.

These shortcomings underscore the inadequacy of single-function support models in facilitating emotional regulation and psychological optimisation for individuals with MCC. At present, major limitations persist in the design of museum environments that aim to serve this population, particularly in terms of care service integration and contextual relevance. These challenges are manifested in the following areas:

1. The design of museum environments often fails to accommodate the specific needs of older adults with MCC, who typically experience increased cognitive load, emotional fluctuations, and the co-occurrence of multiple illnesses. Existing facilities and services are rarely adapted through a systematic, evidence-based approach. The absence of specialized design for sensory stimulation, user-friendly interactive experiences, and tailored guided tours limits accessibility and comfort. In some cases, poorly designed experiences may exacerbate cognitive fatigue or trigger emotional distress, undermining the therapeutic potential of the visit.
2. As cultural service institutions, museums have a responsibility to develop a deeper understanding of the physical, emotional, and cognitive needs of MCC patients—needs that may include cognitive reinforcement, emotional soothing, and opportunities for social interaction. However, current design practices remain largely superficial, lacking in rigorous empirical analysis of user characteristics. This limited understanding leads to environments and service experiences that are misaligned with the actual needs of patients, and thus fail to support emotional regulation or cognitive adaptation effectively.
3. Despite rapid technological advancements, museum environments rarely take full advantage of intelligent design solutions tailored for MCC populations. Existing wayfinding systems, interactive displays, and assistive technologies lack adaptive features or intelligent optimization. There is minimal application of tools such as context-aware sensing, personalized content delivery, or real-time emotional monitoring. As a result, museums miss crucial opportunities to provide precise, personalized support that could significantly enhance the therapeutic and educational impact of the museum experience.

Addressing these challenges requires research to move beyond purely functional considerations and extend into the multidimensional domains of emotion and cognition. A more nuanced analysis is needed to uncover the key factors that shape the museum experience for individuals with multiple chronic conditions (MCC). This involves not only enhancing user satisfaction, but also achieving a comprehensive understanding of their psychological needs, cognitive burdens, and self-regulation mechanisms during museum visits. Accordingly, there is an urgent need to develop innovative theoretical frameworks and methodological approaches capable of systematically investigating and optimising the supportive functions of intelligent environments for MCC populations. Such advancements have the potential to substantially improve both the experiential quality and psychological well-being of these visitors.

This study proposes an innovative approach that centers on the real-world needs of individuals with multiple chronic conditions (MCC) and integrates advanced intelligent design tools to comprehensively enhance their emotional regulation experience within museum settings. The research is committed to a deep and precise exploration of the unique psychological and cognitive needs of this population, using these insights as the foundation for developing an intelligent museum experience design strategy tailored specifically for MCC patients. This strategy aims to support cognitive functioning and emotional regulation through adaptive, technology-enabled environments, thereby improving the overall quality of the museum experience. In doing so, the study seeks to contribute both theoretical advancements and practical guidance for the future development of elder-focused care services within cultural institutions.

To achieve this goal, the study will address the following core research questions:

1. Which environmental design attributes most effectively support emotional regulation and enhance the museum experience for individuals with multiple chronic conditions (MCC)?
2. Based on a patient-centered needs analysis, what are the prioritized preferences of MCC patients across multiple levels of environmental design, and which attributes exert the greatest influence on their emotional and cognitive experience?
3. How can the emotional regulation needs of MCC patients be translated into actionable design features through a designer’s perspective, in order to develop innovative strategies that significantly enhance the therapeutic potential of museum visits?

## 3 Materials and methods

The principal investigator recruited several research volunteers between 20 July 2024 and 15 August 2024 and engaged in extensive communication with the institution through both online and offline methods. This communication aimed at establishing connections with the museum industry and recruiting relevant academic experts, museum design specialists, patients with chronic illnesses, and other participants experienced in museum culture and interactive activities. To safeguard the subjects’ right to informed consent, the researcher distributed a printed informed consent form to patients and secured signatures from both MCC patients and their family members prior to conducting the questionnaire interviews.

### 3.1 Research area

In this study, Nanjing, located in Jiangsu Province and recognized as one of the major cities within the Yangtze River Delta urban agglomeration, was selected as the research site to investigate museum-based experiences and their healing effects on MCC patients. The specific rationale for selecting Nanjing as the research location includes the following aspects:

1. Population and Health Needs: As the capital city of Jiangsu Province, Nanjing possesses abundant cultural resources and supports a large population. By the end of 2023, Nanjing had an estimated resident population of approximately 9.457 million, including around 2.0972 million individuals aged 60 and older. Given the accelerating population aging trend, there is an increasing demand among Nanjing’s elderly population for health-related and public cultural services. According to data from the Nanjing Municipal Health Commission, approximately 42.33% of elderly individuals aged 60 and above in Nanjing exhibit characteristics of MCC. This substantial population of elderly individuals with chronic diseases provides a robust practical foundation and substantial demand for research on museum-based healing space design (33).
2. Economic Development and Infrastructure: Nanjing has sustained consistent economic growth and achieved significant advancements in developing modern infrastructure in recent years. According to the Statistical Yearbook of Jiangsu Province (2023), Nanjing’s GDP growth rate stands out prominently within the Yangtze River Delta region, ranking among the highest in the area. The city’s sustained economic growth provides substantial financial support for developing museums and public cultural infrastructure (34). Additionally, Nanjing hosts numerous world-class cultural and art institutions, such as the Nanjing Museum and the Jiangning Weaving Museum. These museums are well-equipped with advanced facilities and abundant exhibition resources, making them ideal venues for conducting research on recreational and culturally-based healing programs for MCC patients.
3. Policy Support and Public Health Services: The Nanjing Municipal Government places significant emphasis on public health and cultural development, having issued multiple policy directives aimed at enhancing health-related and cultural services for the elderly (35). For instance, the Nanjing 14th Five-Year Plan for the Development of Elderly Services explicitly emphasizes the optimization of elderly health management services and the enhancement of cultural care initiatives. Furthermore, the municipal government prioritizes the specific needs of MCC patients by promoting the development of age-friendly public spaces, fostering the integration of healthcare and cultural resources, and encouraging museums of various types to develop health-focused healing programs. Collectively, these policy orientations provide robust institutional support for conducting the present research.
4. Innovation in Public Culture and Health Services: In recent years, Nanjing has vigorously advanced initiatives aimed at developing a healthy and livable city, continuously fostering innovation within its public cultural service system. For instance, the Municipal Health and Wellness Commission, in collaboration with the Municipal Bureau of Culture and Tourism, has launched a series of cultural and recreational programs specifically designed for the elderly population. By integrating digital technology with traditional cultural resources, museums in Nanjing have progressively explored and implemented healing spaces and health promotion activities tailored specifically for MCC patients. These initiatives provide substantial practical experiences and case studies that significantly contribute to research on integrating recreational therapy with museum space design.

In summary, Nanjing exhibits ideal conditions in terms of population scale, economic development, cultural resources, and policy support for conducting research on museum-based healing space design for MCC patients, thus offering a unique and advantageous research context for this study.Participants will be selected from MCC patients in Nanjing. The data collected from these patients will be used for quantitative analysis and needs assessment, ensuring that the research findings accurately reflect the actual needs of elderly patients with chronic conditions in this region.

### 3.2 Research target

This study focused on patients with MCC, who typically experience organic dysfunction, physiological decline, diminished quality of life, increased treatment burden, heightened mortality risk, and psychosomatic disorders such as anxiety, depression, and loneliness.Participants were recruited from the Department of Geriatric Medicine at Nanjing First Hospital, where the study was conducted in a clinical setting. Clinicians in both outpatient and inpatient wards introduced the study to eligible patients and invited them to participate.

Inclusion criteria were as follows:

1. age ≥ 60 years;
2. clinically diagnosed with two or more chronic diseases (e.g., hypertension, cardiovascular disease, chronic pain, osteoarthritis, chronic obstructive pulmonary disease, or cancer);
3. sufficient communication ability and comprehension to complete the questionnaire; and
4. voluntary participation in the study.

Exclusion criteria included older adults with a history of severe cognitive impairment or psychiatric disorders. Cognitive function was assessed using the Montreal Cognitive Assessment (MoCA) (36), an internationally recognized screening tool for mild cognitive impairment. The MoCA evaluates attention, executive function, memory, language, visuospatial ability, abstract thinking, and orientation, with a total score of 30 points—higher scores indicating better cognitive performance. Participants with MoCA scores below 24 were excluded.

All participants were fully informed of the study’s objectives, procedures, and potential risks before providing written informed consent. The study strictly adhered to the ethical principles of the Declaration of Helsinki, ensuring the protection of participants’ privacy and data confidentiality throughout the research process.

This study conducted a comprehensive screening of patients, with particular attention to issues such as depression, anxiety, and cognitive impairment. Clinical assessments and standardized questionnaires (e.g., GAD-7) were employed to identify and evaluate mental health conditions, ensuring that these factors were adequately considered in the analysis. Common chronic diseases among participants included hypertension, cardiovascular disease, chronic pain, osteoarthritis, chronic obstructive pulmonary disease (COPD), and cancer (37).Among the surveyed elderly patients, 54% had two or more chronic conditions, 28% reported three or more, and 18% suffered from four or more chronic conditions. The prevalence of multimorbidity increased with age, rising from 69% among those aged 65–74 years to 85% among patients aged 88 years and above. Across all age groups, women were slightly more likely than men to experience chronic conditions, with a difference ranging from 1% to 4%.While patients with MCC stemming from different etiologies exhibit varied specific needs, generally, all require enhanced emotional management, psychological support, social connectedness, and improvements in self-worth. Consequently, it is essential to comprehensively address the diverse yet specific demands of MCC patients across various etiologies and design integrated service experiences that effectively meet their collective needs. Through targeted interventions and supportive measures, MCC patients can be assisted in achieving better societal integration and enhanced overall quality of life, as detailed in **Table 2**.

**Table 2:** Age stage table.

Age groups	Characteristics of the period	Manifestations of chronic disease co-morbidity	Demand
60-69 years	Older people in this age group are usually just entering the ageing stage and the incidence of chronic diseases begins to rise gradually.	It is mostly seen in hypertension, diabetes, and osteoarthritis, which are relatively stable but require long-term management.	Social interaction, health education.
70-79 years	The phenomenon of chronic disease co-morbidity is more pronounced in this age group, accompanied by a higher probability of coexistence of multiple chronic diseases and an increased demand for health management.	Coexistence of common chronic diseases such as cardiovascular and respiratory diseases may be accompanied by mild cognitive impairment.	Psychological support, family accompaniment, meaningful activities.
80 years and over	In this age group, patients have the highest rates of chronic disease co-morbidity, often accompanied by multiple co-morbidities, and the health management and care needs of this group are the most complex and urgent.	Multiple chronic conditions are comorbid and prone to acute exacerbations with an increased risk of concomitant disability and cognitive impairment.	Emotional comfort, affectionate companionship, safe and comfortable environment.

### 3.3 Research methodology

This subsection briefly introduces the fundamental concepts of the KANO model integrated with Quality Function Deployment (QFD) and outlines the principles of the PUGH matrix selection method.

#### 3.3.1 KANO model

**Figure 1:**
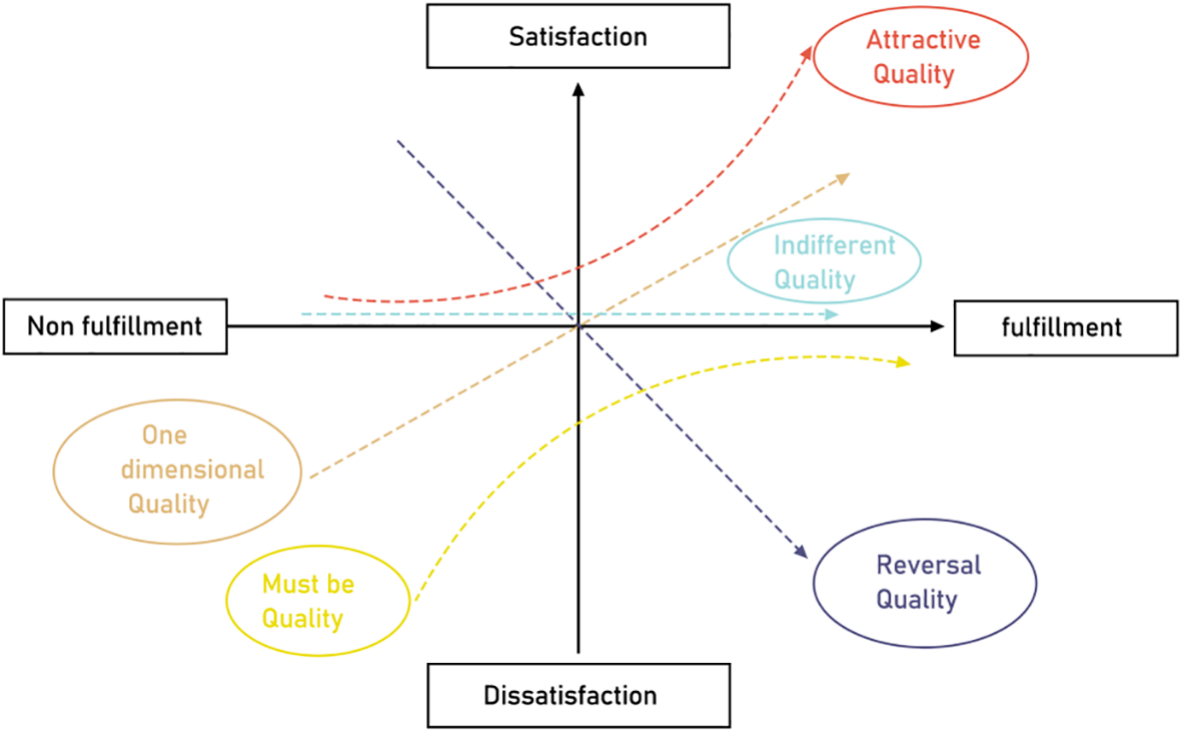
Kano model element relationship.

The primary purpose of the KANO model is to deeply investigate user requirements, categorize and rank these requirements systematically, and evaluate user satisfaction regarding specific product attributes. Additionally, the KANO model analyzes how user requirements influence user satisfaction, reflecting the non-linear relationship between product performance and user satisfaction, as illustrated in **Figure 1** (38).

When a product or service meets Must-be Quality (M) attributes, users typically experience minimal increases in satisfaction; however, if these attributes are not met, user satisfaction sharply declines. For Attractive Quality (A) attributes, fulfillment substantially enhances user satisfaction, whereas their non-fulfillment does not significantly reduce satisfaction. Regarding One-dimensional Quality (O) attributes, fulfilling these attributes moderately increases user satisfaction, while non-fulfillment leads to a noticeable decrease in satisfaction. Indifferent Quality (I) attributes do not influence user satisfaction. Conversely, Reverse Quality (R) attributes negatively impact satisfaction when fulfilled and may increase satisfaction if not fulfilled.

Utilizing the structured approach of the Kano model allows for the effective identification of user attitudes toward products or services and makes explicit customers’ tacit knowledge. This enables targeted optimization of product design or service experiences, thereby more accurately satisfying user expectations and creating additional value beyond those expectations.

#### 3.3.2 Quality function deployment

Quality Function Deployment (QFD), initially proposed by Japanese scholars Yoji Akao and Shigeru Mizuno, is a structured, multilevel deductive analytical method within quality management systems, primarily characterized by the transformation of user requirements into technical specifications. QFD effectively facilitates the mapping of complex relationships between user requirements and technical characteristics. During product development, designers utilize a structured matrix known as the House of Quality (HOQ) to systematically convert user requirements into specific technical features, thereby providing explicit design parameters for the development process (39).

To comprehensively evaluate the design features, we employed the Quality Function Deployment (QFD) methodology, using the KANO model to identify and weight user requirements. Drawing on extensive market research and in-depth interviews, we identified design features aligned with user expectations and constructed a comprehensive evaluation matrix to quantify the correlation between design features and user needs. Taking into account the heterogeneity and weighting of user needs, we quantitatively assessed the strength of correlations and prioritized features with higher scores to guide design decisions, thereby better fulfilling user expectations and enhancing product competitiveness.

#### 3.3.3 Pugh matrix analysis

The Pugh Decision Matrix, developed by Professor Stuart Pugh in Scotland, is a prominent evaluation framework and trade-off analysis tool commonly used in the early stages of product design (40). This method minimizes the occurrence of flawed conceptual designs and enhances the likelihood of generating successful and reliable solutions by supporting qualitative evaluations of design alternatives. The core procedure involves selecting a reference or benchmark solution, qualitatively comparing alternative options against predefined evaluation criteria, and tallying scores to identify the most favorable design through trade-off analysis (41). The Pugh Decision Matrix is valued for its simplicity, practicality, and logical rigor. It effectively decomposes complex, multi-attribute decision problems into hierarchical levels, enabling comprehensive evaluations of each solution’s strengths and limitations across multiple dimensions. This process helps avoid the pitfalls of relying on a single criterion for decision-making. By screening product design requirements at various levels, the method enhances the rationality and consistency of decision-making, ensuring that the final design not only aligns with market demands but also maximizes performance and economic value.

### 3.4 Research process for designing care services for elderly patients with multiple chronic conditions in museums

**Figure 2:**
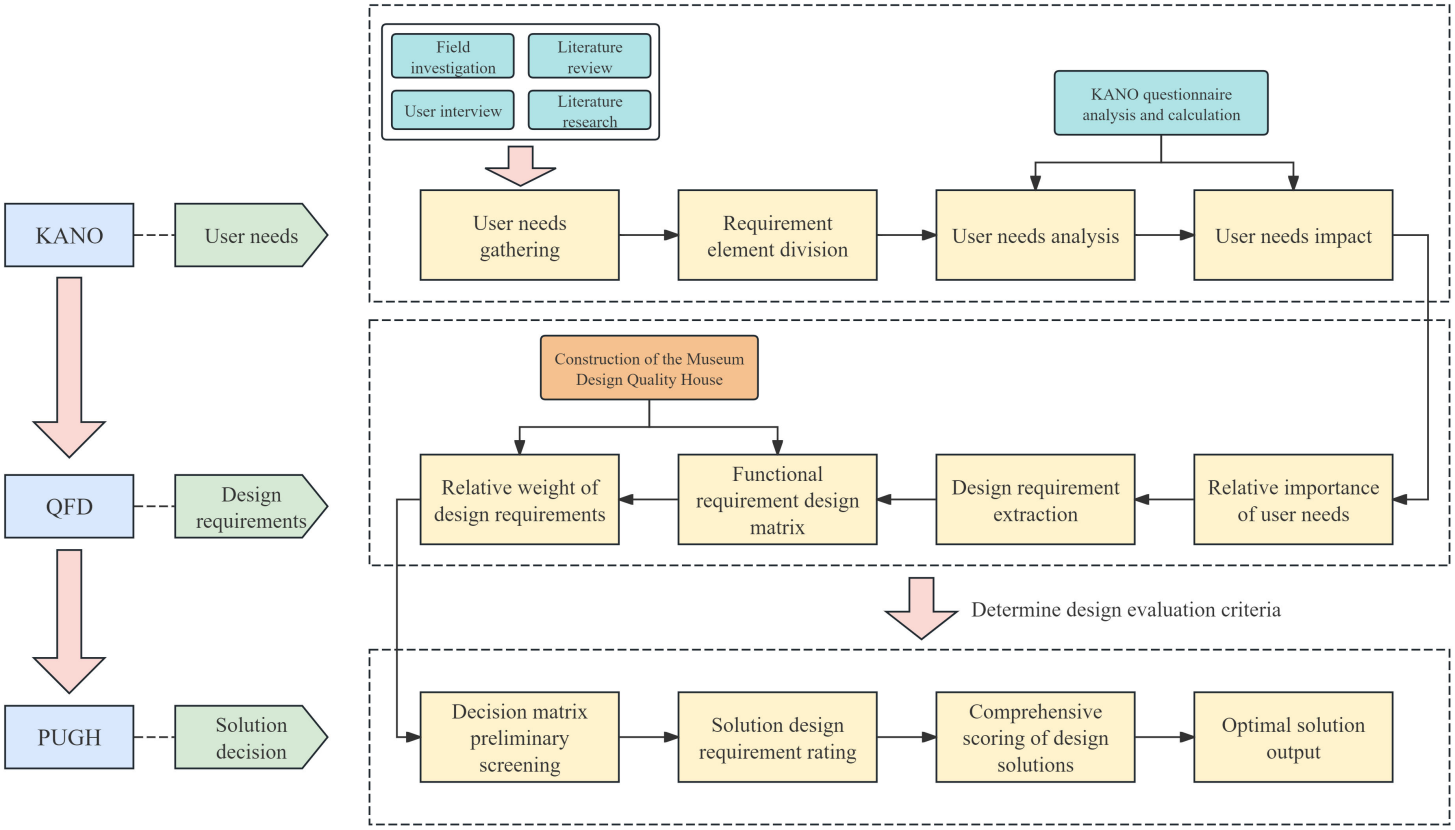
Museum design process based on Kano QFD-PUGH method.

The integrated Kano-QFD-PUGH design methodology spans the entire product development process, encompassing user requirement elicitation, transformation of design requirements, and verification of design scheme decisions. This approach ensures both the accuracy of translating user needs into functional design elements and the methodological rigor in selecting the optimal design solution. Based on the specific product characteristics of this design project, the implementation process is organized into clearly defined stages, as illustrated in **Figure 2**.

1. The Kano model is first employed to analyze user requirements. Questionnaires and user interviews are designed to identify the emotional regulation needs of MCC patients within the museum context, followed by the development of Kano-style questionnaires for in-depth analysis. This enables the classification of user needs into Kano categories and the subsequent calculation and analysis of the relative importance of each need using the Better–Worse coefficient analysis method. This process generates quantitative data on user needs to support the subsequent construction of the comprehensive House of Quality (HOQ).
2. The Quality Function Deployment (QFD) method is used to determine key design elements. First, user need attributes are translated into specific design elements, including spatial layout, sensory stimulation, interactive features, and a sense of emotional belonging within the museum. Second, these design elements are systematically categorized and structured into a matrix of functional design components, and evaluated using the House of Quality (HOQ) to rank their relative importance. This process ensures that the resulting design decisions align closely with user requirements.
3. The Pugh method is applied to conduct program evaluation. First, based on the design requirements, four alternative museum design solutions are identified and subsequently categorized and organized into a design solution matrix. Second, a Pugh decision matrix evaluation is conducted for these alternatives, using a benchmark solution as the reference point for expert scoring and comparative analysis. Ultimately, the optimal design solution is identified by calculating weighted scores.

## 4 Design process for museum experiences tailored to elderly individuals with multiple chronic conditions

### 4.1 Survey and analysis of elderly individuals with multiple chronic conditions

The study consistently incorporated user analysis throughout the design process by integrating desktop research to collate and synthesize information on the current status of the Nanjing Museum and the demographic and behavioral characteristics of the MCC patient group. Subsequently, fieldwork and in-depth interviews were conducted to gain insight into the actual needs of this population. Based on these findings, data from the research and interviews were comprehensively analyzed to develop a categorized list of expected needs across different age groups of MCC patients. The study also identified key pain points encountered by users during museum experiences and synthesized common needs across age groups to inform practical opportunities for future museum experience design. The characteristics of the research population sample are presented in **Table 3**. A total of 162 participants were recruited for this study using a diversified recruitment strategy to ensure both scientific rigor and sample representativeness. The expert evaluation panel consisted of 24 specialists in museum design and 32 academic experts, all of whom possessed more than five years of relevant professional experience. Eligible experts were identified through both open recruitment and recommendation procedures. In addition, all participants had prior knowledge or experience related to healing spaces, ensuring that their assessments were both valid and professionally grounded.For the non-expert group, all participants had prior experience with museums or exhibitions. To guarantee the validity and accuracy of the findings, it was explicitly required that all participants had previously engaged in museum or exhibition activities. These participants generally demonstrated a strong recognition of museum experiences, particularly exhibiting higher needs in areas such as emotional support, social interaction, and self-identity.

**Table 3:** Demographic sample of survey participants.

Classification	Status of experts	Age	Qualifications	Percentage of population
Academic experts	Professor	50-60	PhD Degree	5%
	Associate Professor	41-49	PhD Degree	10%
	Doctoral Student or Researcher	28-38	Master's Degree	5%
Museum Design Specialist	Health and Safety Manager	35-45	Master's degree/doctoral degree	5%
	Design Developer	35-45	Master's degree/doctoral degree	5%
	Experience Interaction Technician	28-35	Bachelor's/postgraduate degrees	5%
Patient	Mild to severe chronic co-morbidities	60-80	/	30%
	Other members of the patient's family	20-50	/	20%
Public	General Visitors	22-50	/	15%

### 4.2 Applying the kano model to analyze the needs of older adults with multiple chronic conditions

The Kano model is a qualitative framework used for analyzing and categorizing user requirements based on their attributes. It is particularly useful for clarifying the relationship between various types of requirements and levels of user satisfaction and is commonly applied in the analysis of personalized user needs. The following outlines the procedure for analyzing the needs of MCC patients using the Kano model:

1. User Needs Acquisition: This study employed a semi-structured interview method to acquire user needs in a direct and efficient manner by probing respondents’ answers in depth, while also encouraging them to propose innovative ideas and provide suggestive feedback (42). During the interview process, participants were first informed that their responses would be associated with museum design and healing environments. The researchers explicitly introduced the concept of a “healing environment” and provided relevant examples to facilitate participants’ understanding of the questions and to support more accurate responses.Based on a predefined interview framework, user needs related to museum design were systematically collected, covering both relatively stable attribute dimensions (e.g., safety) and more variable factors (e.g., behavioral traits), in order to gain a comprehensive understanding of users’ overall demand profiles.
2. Collation of User Needs: The needs expressed by different interviewees may exhibit similarities, inclusiveness, or cross-cutting relationships. Therefore, at the conclusion of the interviews, the acquired needs were organized into a hierarchical structure comprising three levels—functional, usability, and emotional—reflecting the increasing depth of user expectations toward the museum experience.The classification results underwent multiple rounds of discussion and review by the research team’s experts to ensure that each requirement was categorized in accordance with the theoretical framework of the Kano model and to minimize discrepancies in interpretation or classification standards among different researchers. In addition, domain experts were invited to validate the classification of selected requirements, and consistency checking methods were applied to ensure agreement across researchers regarding the categorization of the same requirement. To minimize subjective bias to the greatest extent, all classification procedures strictly followed standardized operational protocols and criteria, ensuring that each requirement was evaluated and categorized through a uniform and systematic process.
3. User Needs Attribution: The User Needs Importance Questionnaire, composed of both positively and negatively phrased items, enables rapid prioritization of user needs. User satisfaction was categorized into five levels: very satisfied, deserved, average, barely acceptable, and dissatisfied. Based on user feedback, requirements were classified into five attribute categories according to the relationship between the degree of fulfillment and user satisfaction, namely: Must-be (M), One-dimensional (O), Attractive (A), Indifferent (I), and Reverse (R) qualities (43).
4. Calculation of Requirement Importance: In some cases, user requirements may simultaneously align with multiple Kano categories, resulting in ambiguous or inaccurate classification. To address this issue, the Better–Worse coefficient is applied as a corrective reference to optimize the Kano model’s classification accuracy. This approach determines the priority weight of each requirement and ensures both the precision of classification and the scientific validity of subsequent design decisions.

### 4.3 Requirement transformation based on quality function deployment

Once the weight of each MCC patient need is derived from the Kano model, these values serve as key reference indicators for evaluating the relevance of user needs to specific design functions, enabling the calculation of a relevance score for each function. Higher scores indicate a stronger alignment between the design function and user needs, thereby enhancing overall user satisfaction.

The needs of MCC patients, identified through the Kano model, were translated into technical design requirements for the museum’s spatial experience services using the Quality Function Deployment (QFD) method (44). The House of Quality (HOQ) forms the core of this process. Serving as a bridge between the ‘voice of the customer’ and the ‘voice of the engineer’, the HOQ visualizes the relationships between user needs and technical specifications (45). Through this process, the absolute and relative weights of each technical feature are calculated, and their interrelationships are analyzed to identify potential contradictions or conflicts during the design process. QFD theory enables the translation of MCC patient needs into functional design elements, with correlation analysis used to assess the degree of association between user needs and design functions. This provides a foundation for developing optimized design solutions that address the needs of MCC patients as well as those of broader museum audiences.

**Figure 3:**
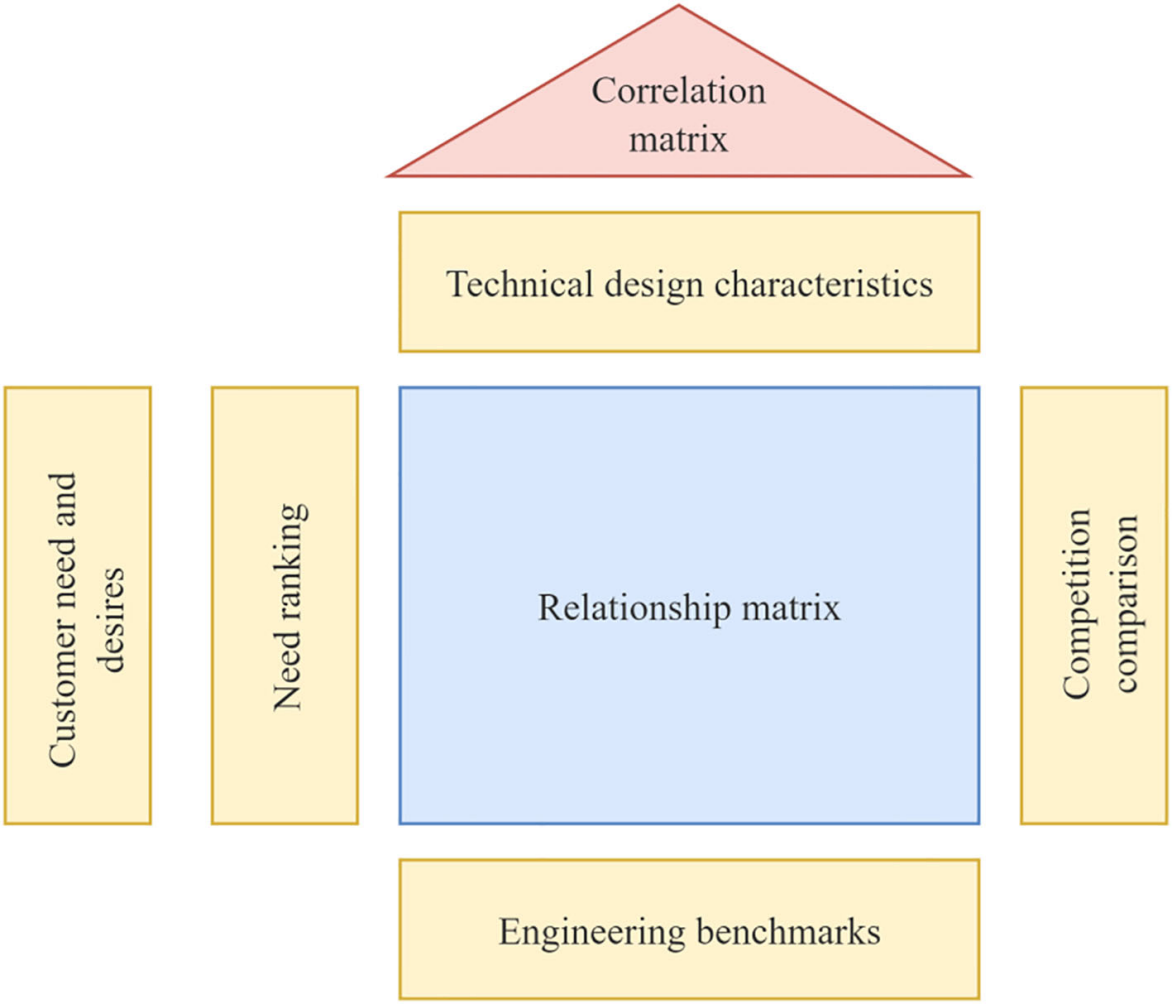
QFD house of quality and basic principle of configuration.

The process of transforming user needs and calculating requirement weights based on the Quality Function Deployment (QFD) method is outlined as follows:

1. Quality Planning of User Demand Elements: The refinement of user demand importance involves two key components: market competitiveness assessment and quality target planning. The former enables designers to identify differentiation points in benchmark products and clarify directions for product development and refinement, while the latter supports designers in conducting quantitative analyses of quality elements, which form the basis for evaluating user requirement importance within the QFD framework. Focusing on key demand elements, a quality attribute evaluation questionnaire was administered to museums with mature market designs, and the results were used as a key input to refine the importance weights of needs specific to chronically ill users.
2. Conversion of Design Requirements into Functional Elements: A dedicated technical team for museum spatial experience services was established. Based on the primary user needs identified through the Kano model, QFD theory was applied, and expert evaluations were conducted by practitioners with relevant experience, focusing on the identification and assessment of functional design elements. The resulting design function list encompasses all functional components derived from the needs of chronically ill patients as well as other museum visitors.
3. Construction of the House of Quality (HOQ): The left side of the HOQ contains the weighted user needs of MCC patients, which are calculated using the Kano model and recorded in the user needs weighting section. The ceiling of the HOQ displays the museum spatial engineering attributes designed to meet the needs of various MCC patients, and the technical characteristics corresponding to each user need are analyzed and mapped accordingly. The correlation matrix located in the roof section represents the interrelationships among the museum’s spatial engineering attributes. The right wall of the HOQ presents a comparative analysis of design alternatives proposed by designers and technical experts, focused on quality improvement priorities. The “floor” section of the HOQ summarizes the weighted evaluation scores based on the overall design relevance and technical assessment values, as illustrated in **Figure 3**.

### 4.4 Pugh selection matrix

The PUGH Decision Matrix is an efficient and practical qualitative decision-making tool that enables decision-makers to systematically evaluate the advantages and disadvantages of complex and dynamic design scenarios, thereby facilitating the accurate identification and selection of the optimal solution.

1. Initial Screening of Design Solutions: Based on the importance rankings of functional elements derived from the House of Quality (HOQ) analysis, the design team developed a set of preliminary design solutions. Each functional element served as an evaluation criterion for scoring all proposed designs. During the scoring process, expert opinions, user needs, and technical feasibility were comprehensively considered to ensure the reliability and validity of the evaluation. A detailed scoring matrix was constructed to present the performance of each design solution across all functional elements, thereby providing a robust foundation for subsequent decision-making and solution refinement.
2. Comprehensive Evaluation of Design Solutions: Following the initial screening, a more in-depth evaluation was conducted using the PUGH matrix to comprehensively analyze each design solution. The competitiveness of each solution was assessed across all functional elements, with individual scores compiled and weighted to derive an overall performance score for each design proposal. Based on these scores, the highest-performing solutions were shortlisted for the next phase of detailed design development and optimization. This process not only identifies the most promising design candidates but also highlights critical areas for further enhancement.
3. Satisfaction Assessment: A seven-point Likert scale was employed as the primary measurement tool, with data collection combining experimental scenario experiences and questionnaire surveys. This approach ensured both contextual authenticity and the acquisition of quantifiable user feedback. For data analysis, descriptive statistics were used to present overall trends, reliability testing was conducted to ensure the internal consistency of the scale, and variance analysis together with correlation analysis were applied to examine differences and associations between design alternatives and user characteristics. By integrating the quantitative decision-making outcomes of the Pugh matrix with subjective user satisfaction feedback, this study balanced scientific rigor with user perspectives during the screening and optimization of design alternatives, thereby ensuring that the final design was both empirically grounded and aligned with the actual needs of the target population.
4. Final Analysis and Selection of the Museum Experience Design Solution: Following comparative analysis and multiple rounds of refinement, the design team finalized the most suitable solution based on the evaluation outcomes of the PUGH matrix, integrated with insights from complementary design methodologies and technical tools. The selected solution not only satisfies the original user requirements but also achieves an optimal balance among quality, performance, and feasibility. Designed to be both functional and sustainable, the final solution aims to deliver a high-quality user experience in practice and to enhance the museum visit and therapeutic experience for MCC patients.

## 5 Results

### 5.1 Analysis of findings

**Figure 4:**
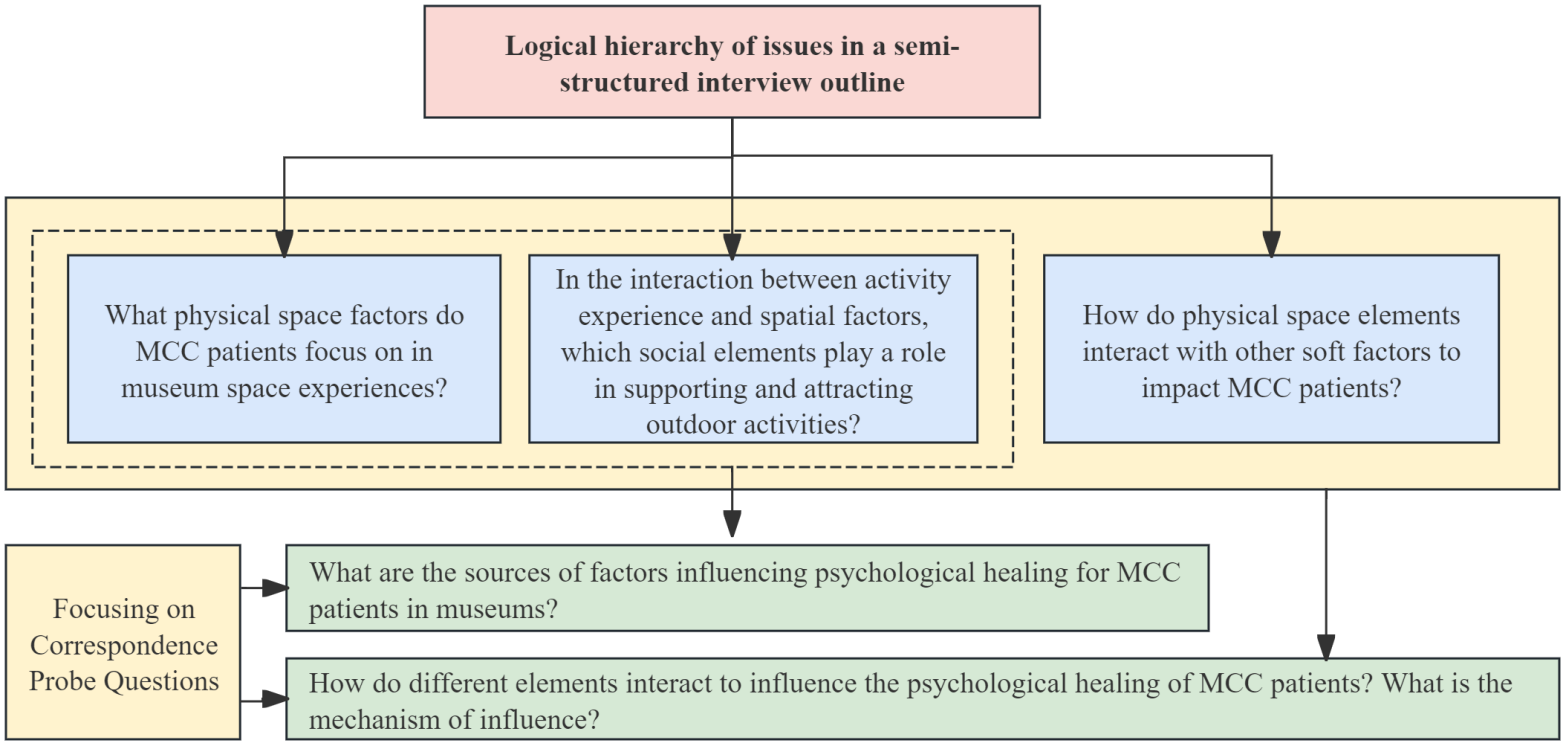
Semi-structured interview outline and probe question correspondence.

To ensure the accuracy and representativeness of the research data, this study centers MCC (Multiple Chronic Conditions) patients as the primary research subjects. The survey scope extends to related stakeholder groups, including medical and nursing staff from hospitals and rehabilitation centers, patients’ family members, and other relevant individuals. The interview outline and associated probing questions are presented in **Figure 4**. This study was approved by the institutional ethics committee and carefully accounted for the potential cognitive or sensory impairments among the MCC population. To address these concerns, concise explanations and adapted materials (e.g., large-font, high-contrast text) were provided in questionnaires and interviews. When necessary, family members or caregivers were permitted to accompany participants to ensure full comprehension and voluntary participation. These measures safeguarded the ethical integrity of the study and enhanced the validity of the findings.The study systematically categorizes the design elements of museum services that influence the psychological healing of MCC patients into two dimensions: hard elements and soft elements. Among these, physical space—as the principal source of hard elements—is analyzed in terms of its influence mechanisms on patients’ psychological healing. This is achieved by deconstructing the museum’s spatial composition, representational features, and environmental attributes.At present, research on the role of spatial elements in supporting the psychological healing of MCC patients remains in an exploratory phase. Existing studies primarily examine core dimensions such as infrastructure configuration, environmental aesthetics, and safety systems. Empirical evidence has shown a positive correlation between museum spatial design and the psychological well-being of MCC patients (46). This relationship is reflected in several critical factors, including the sophistication of infrastructure, the professionalism of ancillary services, the inclusiveness of spatial culture, and the reliability of environmental safety systems. Collectively, these elements contribute to enhancing patients’ visiting experiences, promoting social engagement, and improving mental health outcomes.

The study adopted grounded theory as the primary analytical framework (47). A total of 157 valid interview transcripts—comprising approximately 70,000 words—were analyzed through a structured three-level coding process. At the methodological level, the study innovatively introduced the analytical dimension of Perceived Environment Attributes (PEA), which complements the traditional focus on Objective Environment Attributes (OEA). This addition addresses the underrepresentation of users’ subjective experiences in spatial design evaluation, enabling a more comprehensive understanding of user-environment interactions.The analysis of soft elements specifically focused on the mechanisms through which the social support system influences user well-being. Particular attention was paid to environmental support roles such as emotional reinforcement, interactive tools, and service experiences. However, the coupling mechanisms between these social elements and specific spatial configurations remain an area for further investigation.The grounded theory analysis strictly followed the procedural logic of “labelling-conceptualisation-categorisation.” Ultimately, the study constructed a hierarchical framework: the first-level classification consists of hard and soft elements, while the second-level classification further distinguishes spatial elements into three subcategories and social elements into another three categories, as presented in **Table 4**.

**Table 4:** Qualitative extraction of the elements of influence (spatial and soft elements) of the museum space on the psychological healing of MCC patients.

Category of elements (level 1)	Category of elements (level 2)	Corresponding subset
Hard elements - Physical space	Museum infrastructure (connotation: the spatial components that are essential for the fulfilment of the visitor experience, the physical media that are directly involved in the behaviour that takes place).	Exhibition spaces, guided tours and information service areas, streamlined movement design, integration of natural elements, and sustainable design.
	Auxiliary service type facilities (Context: Facility elements that provide ancillary experiential services)	Outdoor extension space, barrier-free facilities, colourful atmosphere creation, etc.
	Spatial cultural elements (connotation: spatial components such as cultural facilities)	Nursing knowledge science, restorative art creation, etc.
Soft factors - Social factors	Emotional comfort support (connotation: emotional comfort from social interactions such as care, trust and love to support their activities to take place)	Remote Participation, Emotional Balance Games, Multi-sensory Healing Experiences, Dynamic Showroom, Feedback Mechanisms, Thematic Contextual Experiences, Sound Healing, Positive Suggestion, and more.
	Interactive instrumental support (connotation: the provision of interactive equipment and services, i.e., the use and functioning of site facilities by the subject, encompassing the process of active excavation and adaptation of the space by the human being)	Visual information, wearable device support, personalised guided tours, exhibit interaction, creative workshops and experimental areas, etc.
	Experiential support for services (connotation: access to information that guides and feeds back on one's own behaviour and facilitates the formation of long-term behavioural patterns)	Health data tracking, health self-testing, AI health advisor, health behaviour guidance, feedback technical support, etc.

**Figure 5:**
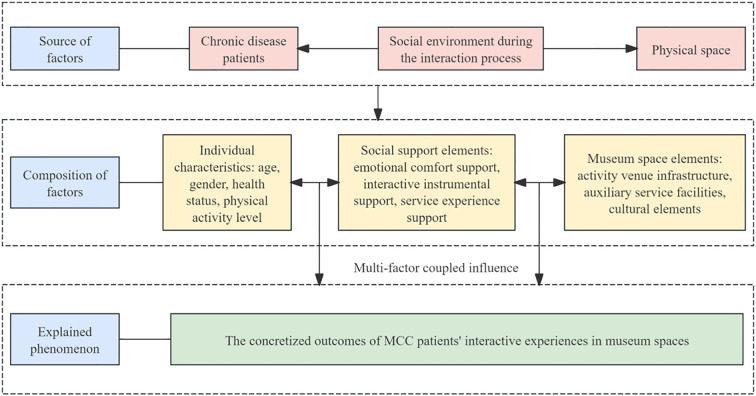
Compositional interpretation of impact mechanisms.

**Figure 5** illustrates the mechanism through which museum experiences contribute to the psychological healing of patients with MCC. This theoretical framework was derived from the coding procedures of constructivist grounded theory analysis. By integrating analyses of perceptual spatial elements and the social support system, the framework was systematically developed.

Following the three-level coding system of grounded theory, the analysis proceeded as follows: During the open coding stage, raw textual data (e.g., interview transcripts) were analyzed line by line and paragraph by paragraph to identify key words or concepts. In the axial coding stage, similar or related concepts were integrated to form higher-order categories. During the selective coding stage, a single core category was identified from the major categories, and the relationships between this core category and other categories were established, thereby constructing the theoretical framework or model.

The entire coding and analytical process was facilitated by NVivo 12 software. Two researchers independently performed the coding, and inter-coder reliability was assessed (Cohen’s Kappa > 0.80) to ensure the credibility and reproducibility of the analysis.

The psychological healing effect experienced by MCC patients within the museum environment is conceptualized as the outcome of a complex interactive experience. The various forms of engagement and activity observed throughout this process represent concrete dimensions through which this psychological transformation manifests.The core components of this mechanism are derived from the dynamic interaction among three influencing subjects. First, the individual characteristics of MCC patients determine the variability in their psychological responses to the environment. Second, perceived space acts as the primary medium of interaction between the individual and the physical setting, mediating their sensory and emotional experience. Third, social support factors serve as soft relational ties that connect patients to the environment, functioning as intermediary agents that regulate and enhance the interaction between the individual and the spatial setting.This theoretical framework systematically explains how the museum’s spatial environment contributes to the psychological recovery of MCC patients through multiple interrelated pathways involving personal, spatial, and social dimensions.

### 5.2 Analysis of results of user satisfaction indicators

1. User demand acquisition:

The research team conducted a systematic collection of relevant data and applied the KJ method to categorize demand attributes and define functional indicators. During this process, redundant demands and invalid functional elements were eliminated, resulting in the development of an initial functional requirements list, as shown in **Table 5**. It should be noted that the terminology in this list does not directly reflect the participants’ original expressions but rather represents the outcomes refined and conceptualized by the researchers through guidance, abstraction, and synthesis.

**Table 5:** Initial user requirements of museum.

Serial number	Requirement item	Serial number	Requirement item	Serial number	Requirement item
1	Accessibility	9	Emotional Balance Game	17	AI Health Advisor
2	Exhibit Interaction	10	Positive Psychological Cues	18	Dynamic Showroom
3	Colour Atmosphere Creation	11	Multi-sensory Experience	19	Streamlined Dynamic Design
4	Personalised Guided Tours	12	Integration Of Natural Elements	20	Thematic Contextual Experiences
5	Outdoor Extension Space	13	Feedback Mechanisms	21	Nursing knowledge science
6	Visualisation	14	Sustainable Design	22	Virtual Healing Experience
7	Wearable Device Support	15	Health Data Tracking	23	Health Behaviour Guidance
8	Remote Participation	16	Health Self-Test	24	Restorative Art Creation

2. User requirements collation

The project team conducted a hierarchical classification of the functional cards derived from **Table 5**. First, all functional requirements on the cards were categorized according to their objective relationships, resulting in the formation of secondary functional indicators. Subsequently, the research group’s experts synthesized and consolidated these secondary indicators to extract higher-level representative requirements, thereby generating the primary demand indicators. The development of the primary indicators was carried out through collective discussion, inductive refinement, and review by the research team members, ensuring that each primary indicator encompassed multiple secondary functional indicators and effectively reflected the museum experience design needs of MCC patients. This process ultimately produced a structured list of functional requirements for the museum experience design tailored to MCC patients, as presented in **Table 6**.

**Table 6:** Functional requirements analysis table.

Level 1 requirements	Serial number	Functional indicators	Function
Physical Environment Support	Q1	Accessibility	Provide barrier-free access and facilities for chronic co-morbidities with mobility impairments to ensure a smooth visit.
	Q2	Colour Atmosphere Creation	A soft colour palette is used to help chronic co-morbidities relieve anxiety and psychological stress.
	Q3	Outdoor Extension Space	An outdoor healing area is set up to allow patients to relax through nature contact.
	Q4	Integration Of Natural Elements	Natural elements such as greenery and running water are introduced to create a soothing healing environment for patients.
	Q5	Sustainable Design	Adoption of environmentally friendly materials and energy-saving technologies to reduce the impact on the environment and create a healthy and safe visiting environment for patients with chronic co-morbidities.
	Q6	Streamlined Dynamic Design	Optimising the tour route to reduce physical exertion and improve comfort for patients with chronic co-morbidities.
Interactive Experience	Q7	Exhibit Interaction	Design interactive exhibits that are easy to use and help patients gain knowledge in a relaxed experience.
	Q8	Personalised Guided Tours	Tailor the visit to the patient's health status and interests to optimise the individual experience.
	Q9	Wearable Device Support	Patients can use the device to trigger dynamic content, personalised tasks or engage in real-time interactive activities, making the healing process more fun and immersive.
	Q10	Remote Participation	Provide online visit options for patients with limited mobility to heal remotely.
	Q11	Dynamic Showroom	Showrooms can create a soothing or active atmosphere to enhance the healing effect and participatory experience.
	Q12	Thematic Contextual Experiences	Help patients relieve physical and mental exhaustion through immersive situations (e.g., forests, oceans).
Mental and Emotional Healing	Q13	Emotional Balance Game	Interactive games to help people with chronic co-morbidities identify and regulate their emotions.
	Q14	Positive Psychological Cues	Showcase positive cases and positive messages to boost patients' confidence in recovery.
	Q15	Multi-Sensory Experience	Combining the senses of sight, sound, and smell to provide patients with holistic sensory healing.
	Q16	Virtual Healing Experience	Using AR and VR technology, we simulate natural scenes to provide immersive healing for patients.
Health Management Support	Q17	Health Data Tracking	Real-time monitoring of patient health data, providing personalised feedback to support health management.
	Q18	Health Self-Test	Set up a self-help health assessment tool to help patients understand their situation and get advice.
	Q19	AI Health Advisor	Real-time health counselling through AI to support health decision-making for patients with chronic co-morbidities.
	Q20	Health Behaviour Guidance	Help patients learn and establish healthy habits to improve chronic disease co-morbidity management.
Education and feedback mechanisms	Q21	Visualisation	Health knowledge is presented visually through dynamic charts and graphs, making it easy for patients to understand and apply.
	Q22	Feedback Mechanisms	Collecting feedback on the patient visit experience and providing personalised health improvement advice.
	Q23	Nursing Knowledge Science	Provide patients and families with knowledge of chronic disease co-morbid care to enhance quality of life.
	Q24	Restorative Art Creation	Provide opportunities for artistic creativity to help patients express their emotions and relieve stress through art.

3. User demand attribution

This study investigates the demand attributes of museum experience design for MCC patients in the current market context. To capture user preferences and perceptions, a questionnaire was designed from two dimensions: functional items and interactive experience. A five-point Likert scale was applied (48), offering the following response options for each item: very unfavorable, unfavorable, neutral, favorable, and very favorable. Each response was assigned a score ranging from 1 to 5, enabling quantitative assessment of user attitudes from both positive and negative perspectives. This approach supports the identification of users’ needs and emotional responses. The detailed structure of the questionnaire is shown in **Table 7**. The questionnaire evaluated 24 items related to the museum experience of MCC patients. These items were jointly developed by museum experts, psychologists, and researchers, and subsequently validated by experts. The questions were specifically oriented toward healing objectives, avoiding overly general museum features; instead, the generation of items explicitly emphasized their relevance to the needs of MCC patients. To ensure the validity of the assessment, participants’ responses were strictly focused on the healing functions of museums, thereby minimizing the potential influence of general or stereotypical impressions of museums on their answers.

**Table 7:** Five-point likert scale.

Requirements project options					
Provision Of Requirement	Very unlikeable-1	No favorable impression-2	No sensation-3	Favorable impression-4	Very favorable impression-5
No Provision of Requirement	Very unlikeable-1	No favorable impression-2	No sensation-3	Favorable impression-4	Very favorable impression-5

Before analyzing the collected user requirement data, a Kano model evaluation table was constructed. According to Kano’s framework, user requirements are categorized into five distinct types based on their impact on user satisfaction: Must-be (M), One-dimensional (O), Attractive (A), Indifferent (I), and Reverse (R) requirements. Each category reflects a different performance-satisfaction relationship. The classification is based on the response patterns to paired positive and negative questions, and the correspondence between these responses and Kano requirement types is detailed in **Table 8**.

**Table 8:** Kano evaluation form.

User needs		Reverse problem				
		Very favorable impression-5	Favorable impression-4	No sensation-3	No favorable impression-2	Very unlikeable-1
Forward Issue	Very favorable impression-5	*Q*	*A*	*A*	*A*	*O*
	Favorable impression-4	*R*	*I*	*I*	*I*	*M*
	No sensation-3	*R*	*I*	*I*	*I*	*M*
	No favorable impression-2	*R*	*I*	*I*	*I*	*M*
	Very unlikeable-1	*R*	*R*	*R*	*R*	*Q*

The questionnaire was distributed online, yielding a total of 162 responses. After excluding six invalid questionnaires—due to incomplete responses, unusually short completion times, or identical answer patterns across multiple items—a total of 156 valid responses were retained for analysis. Based on the survey results and in reference to the Kano evaluation table, the demand attributes were classified accordingly. The summarized classification of user requirements for museum experience design tailored to MCC patients is presented in **Table 9**.

**Table 9:** Classification of Kano attributes for design requirements.

Serial number	*A*	*O*	*M*	*I*	*R*	Causality
*Q*1	22	16	58	40	20	M
*Q*2	36	1	28	60	31	I
*Q*3	16	11	31	44	54	R
*Q*4	20	61	20	44	11	O
*Q*5	10	15	64	37	30	M
*Q*6	21	16	50	39	30	M
*Q*7	16	12	30	70	28	I
*Q*8	42	9	25	57	23	I
*Q*9	55	31	21	33	16	A
*Q*10	20	20	23	27	66	R
*Q*11	27	58	10	46	15	O
*Q*12	30	55	19	38	14	O
*Q*13	62	13	25	38	18	A
*Q*14	19	60	12	43	22	O
*Q*15	16	67	7	45	21	O
*Q*16	24	56	9	48	19	O
*Q*17	57	10	31	38	20	A
*Q*18	36	17	31	52	20	I
*Q*19	53	22	27	43	11	A
*Q*20	28	18	64	34	12	M
*Q*21	20	16	61	45	14	M
*Q*22	21	26	64	32	13	M
*Q*23	31	29	29	53	14	I
*Q*24	59	26	23	33	15	A

4. Calculation of the importance of demand:

Based on the demand attributes summarized from the Kano questionnaire, the results were further analyzed using the Better–Worse Index method to establish the relationship between each demand attribute and its influence on user satisfaction. The Better–Worse Coefficient was introduced as a correction mechanism to adjust the preliminary Kano classification results and to determine the priority weight of each user requirement.In this framework, S_i_ represents the Better Coefficient, which reflects the positive impact of fulfilling a specific user need on overall satisfaction. Conversely, D_i_ denotes the Worse Coefficient, capturing the negative impact of not meeting the corresponding requirement. The mathematical expressions for these coefficients are defined in Equations 1 and 2.


(1) $$S_{i}=\frac{A_{i}+O_{i}}{A_{i}+O_{i}+M_{i}+I_{i}}$$

(2) $$D_{i}=(-1)\times \frac{M_{i}+O_{i}}{A_{i}+O_{i}+M_{i}+I_{i}}$$

The questionnaire was designed to quantitatively evaluate 24 need-oriented items related to the museum experience of MCC patients. After data collection, the responses were imported into SPSS for reliability and validity testing to assess the consistency and structural soundness of the instrument. The validation results are presented in **Table 10**. In terms of reliability, the Cronbach’s alpha (49) (Cronbach.α) coefficients for the overall questionnaire, as well as for the positively and negatively phrased items, all exceeded the accepted threshold, indicating high internal consistency. These results confirmed both the credibility and structural validity of the instrument. In the subsequent quantitative analysis, the importance of each of the 24 need items will be calculated and ranked based on the 156 valid questionnaire responses (50).

**Table 10:** Results of questionnaire reliability test.

Test item	Formula	Cronbach.α	Reference threshold
Forward Issue Cronbach.alpha coefficient	$\alpha =\frac{N}{N-1}\left[1-\frac{\sum s_{I}^{2}}{s^{2}}\right]$ (3)	0.86	0.76
Reverse question Cronbach.alpha coefficient		0.72	0.62
Questionnaire as a whole Cronbach.alpha coefficient		0.83	0.70

The Better coefficient in Equation (1) reflects the degree of user satisfaction associated with fulfilling a specific functional requirement—specifically, the first design function in this case. Conversely, the Worse coefficient in Equation (2) measures the degree of user dissatisfaction when that requirement is not met. The variables in the formula represent the percentage of users who classified each function under the four Kano requirement categories: Attractive (A), One-dimensional (O), Must-be (M), and Indifferent (I) (51).Based on these equations, the data for the 24 functional design requirements were substituted into the formulas, and the calculated results are presented in **Table 11**.

**Table 11:** Results of the analysis of the Better-Worse index for each indicator.

Serial number	*S_i_*	*D_i_*	Serial number	*S_i_*	*D_i_*
*Q*1	0.28	-0.54	*Q*13	0.54	-0.28
*Q*2	0.30	-0.23	*Q*14	0.59	-0.54
*Q*3	0.26	-0.41	*Q*15	0.61	-0.55
*Q*4	0.56	-0.56	*Q*16	0.58	-0.47
*Q*5	0.20	-0.63	*Q*17	0.49	-0.30
*Q*6	0.29	-0.52	*Q*18	0.39	-0.35
*Q*7	0.22	-0.33	*Q*19	0.52	-0.34
*Q*8	0.38	-0.26	*Q*20	0.32	-0.57
*Q*9	0.61	-0.37	*Q*21	0.25	-0.54
*Q*10	0.44	-0.48	*Q*22	0.33	-0.63
*Q*11	0.60	-0.48	*Q*23	0.42	-0.41
*Q*12	0.60	-0.52	*Q*24	0.60	-0.35

**Figure 6:**
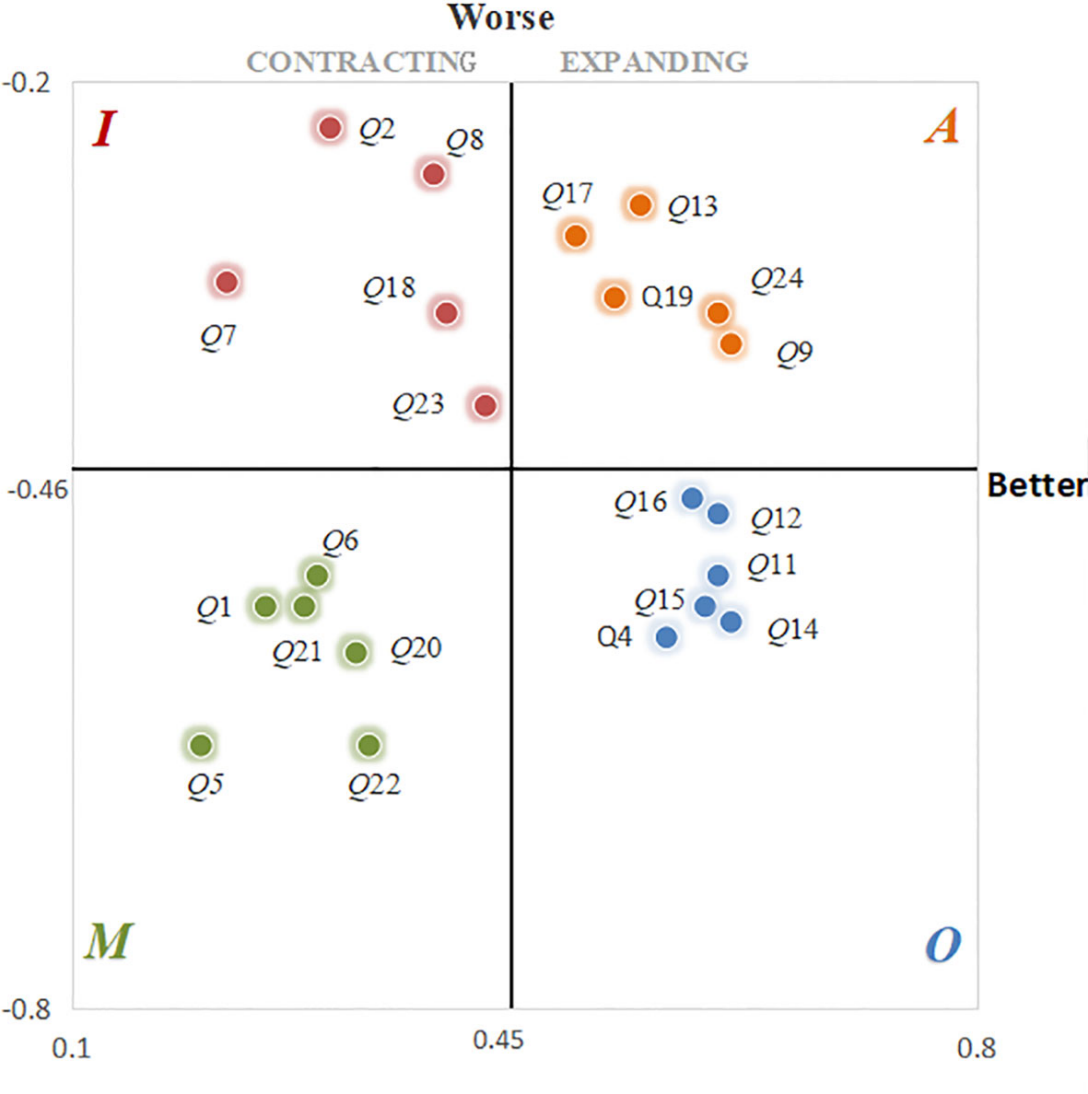
Four-quadrant diagram.

To more clearly illustrate the importance of each museum design requirement from the user’s perspective, a four-quadrant diagram was constructed to plot the Better and Worse index values for each functional attribute (52). This visualization enables the functional requirements to be categorized based on their contribution to user satisfaction and dissatisfaction. The resulting distribution allows for an intuitive division of the requirements into distinct attribute types, as presented in **Figure 6**.

From the Better value in the figure Q4, Q11, Q12, Q14, Q15 and Q16 have significant impact on satisfaction improvement. Q4 creates a soothing healing environment for patients by introducing natural elements such as green plants and flowing water.Q11 creates a soothing or active atmosphere to enhance the healing effect and participation experience.Q12 helps patients to relieve their physical and mental fatigue by means of immersive contexts (e.g., forests, oceans).Q14 features the display of positive cases and positive energy messages to enhance patients’ confidence in recovery.Q15 focuses on the combination of visual, auditory, olfactory, etc., to provide patients with comprehensive sensory healing.Q16 uses AR and VR technology to simulate natural scenes to provide patients with immersive healing.These requirements, based on their significant impact identified in the Kano analysis, will be incorporated as priority requirements into the subsequent QFD analysis.

Based on the Kano model classification of museum design demand attributes from the previous stage, the influence weights of key demand elements were calculated using the formula presented in Equation 4. The results of this calculation are summarized in **Table 12**.

**Table 12:** Impact results of Kano demand analysis.

Demand	*S_i_*	*D_i_*	*ω_i_*	Weight order
*Q*4	0.56	-0.56	0.179	1
*Q*11	0.60	-0.48	0.169	5
*Q*12	0.60	-0.52	0.169	4
*Q*14	0.59	-0.54	0.173	3
*Q*15	0.61	-0.55	0.176	2
*Q*16	0.58	-0.47	0.164	6


(4) $$\omega_{i}=\max\left\{\frac{s_{i}}{\sum s_{i}},\left|\frac{D_{i}}{\sum D_{i}}\right|\right\}$$

### 5.3 An analysis of museum design elements based on the QFD model

1. Quality planning for user requirement elements

To comprehensively assess the quality level of existing museum designs and identify potential areas for improvement, this study conducted an investigation and analysis of several museums in the market. This process clarified the key domains and quality benchmarks of current museum design, which were subsequently used to establish target quality standards for the present design planning. Specifically, we carried out a quantitative analysis of demand elements related to the spatial service experience of museums, selecting three representative comprehensive historical and art museums as research subjects: the Palace Museum (G), the Nanjing Museum (N), and the Hebei Museum (H). This selection ensured broad coverage and representativeness of the sample, encompassing museum design practices across different regions and cultural contexts.The respondents consisted of museum designers and professionals from related industries. A five-point rating system was employed (1 = poor, 2 = fair, 3 = moderate, 4 = good, 5 = excellent) to evaluate museum design quality, thereby ensuring the reliability of the collected data. Based on these data, the study further calculated the improvement ratio(Ri) of user demand importance to perform a quantitative analysis of design elements across the selected museums. This process facilitated the identification of the most influential design elements and provided direct empirical support and theoretical grounding for the optimization of the Nanjing Museum’s design. Ultimately, through the application of the QFD model, we were able to more precisely determine the degree of alignment between museum design elements and the emotional regulation needs of MCC patients, thus offering a scientific basis for subsequent design decisions.


(5) $$R_{i}=\frac{Q_{b}}{Q_{a}}$$

In Equation 5, *Q_a_* represents the mean value of the current design quality for each demand element, while *Q_b_* denotes the corresponding target quality level. These values are derived from the expert evaluation results described earlier. The outcomes of the questionnaire survey and the calculated improvement rates using Equation (5) are summarized in **Table 13**.

**Table 13:** Need element quality planning.

Demand	Market research			Q_a_	Q_b_	R_i_
	*G*	*N*	*H*			
*Q*4	3	3	2	3	3	1
*Q*11	3	3	1	2	2	1
*Q*12	4	3	3	3	4	1.3
*Q*14	2	2	2	2	3	1.5
*Q*15	4	3	2	3	3	1
*Q*16	2	3	2	2	3	1.5

2. Design Requirements and Functional Elements Conversion

To ensure the scientific validity and reliability of the proposed design features, a professional assessment team was assembled, comprising two categories of experts: senior museum designers with more than five years of experience in exhibition and spatial planning, and psychologists with a background in clinical psychology who specialize in environmental psychology and healing space research. This interdisciplinary team collaborated to review, validate, and define the experiential design features of the museum, ensuring that they align with the core needs of both MCC patients and general visitors.Drawing on the established correspondence between user needs and technical design attributes, the study conducted a systematic analysis of each functional requirement. Particular emphasis was placed on the unique needs of MCC patients. Through iterative expert consultation and integration, the design requirements were refined and consolidated into a comprehensive summary of museum experience service features, as presented in **Table 14**. The design requirements have all been validated by existing empirical studies, ensuring the scientific rigor and reliability of each requirement.

**Table 14:** Correspondence table between user requirements and technical characteristics.

Level 1 requirements	Serial number	Functional indicators	Serial number	Technical characteristics
Physical Environment Support	*Q*4	Integration Of Natural Elements	*S*1	Indoor Greening System (53)
			*S*2	Bionic Morphological Design (54)
			*S*3	Natural light and shadow simulation (55)
Interactive Experience	*Q*11	Dynamic Showroom	*S*4	Interactive Dynamic Devices (56)
			*S*5	Digital Projection Mapping (57)
	*Q*12	Thematic Contextual Experience	*S*6	Virtual Robot Guidance (58)
			*S*7	Narrative Spatial Sequences (59)
Mental and Emotional Healing	*Q*14	Positive psychological Cues	*S*8	Positive visual symbols (60)
			*S*9	Colour Psychology Applications (61)
	*Q*15	Multi-Sensory Experience	*S*10	Soundscape system design (62)
			*S*11	Tactile Interactive Interface (63)
			*S*12	Odour diffusers (64)
	*Q*16	Virtual Healing Experience	*S*13	VR Meditation Space (65)
			*S*14	Digital Art Healing (66)

3. HOQ Construction

The construction of the House of Quality (HOQ) is the core component of the Quality Function Deployment (QFD) process. Its primary function is to systematically map and translate user needs—particularly those of chronically ill patients—into corresponding design function elements. This translation enables the prioritization of design attributes, ensuring that quality control in museum design aligns with user-centered goals.In the context of museum spatial experience service design, the HOQ is constructed through the following steps:

The first step involves constructing the left wall of the House of Quality, which represents the prioritized user requirements. Based on the results of the Kano model analysis, user-centered attribute requirements were screened, and six key requirements—Q4, Q11, Q12, Q14, Q15, and Q16—were selected due to their strong influence on user satisfaction. The corresponding attribute descriptions and their calculated importance weights were then imported into the HOQ to populate the left wall.

In the second step, the ceiling and roof of the House of Quality are constructed. First, the key user requirements (Q4, Q11, Q12, Q14, Q15, Q16), previously identified through Kano analysis and listed in **Table 14**, are reorganized according to the principles of design engineering advancement. These requirements are then translated into corresponding functional elements of the museum spatial experience service and imported into the HOQ to form its ceiling, which represents the technical features that address user needs.Next, a pairwise correlation analysis is conducted among the functional elements. For each pair, a symbol is used to indicate the nature of their relationship: a ‘+’ denotes a positive correlation, while a ‘−’ indicates a negative correlation. This correlation matrix constitutes the roof of the HOQ and provides insight into potential synergies or conflicts between design features.

To represent the strength of the relationships between user requirements and functional elements in the House of Quality (HOQ), three symbols are used: ⊚,○, and ⋄, corresponding to weight values of 5, 3, and 1, respectively. These symbols denote strong, medium, and weak correlations and are used to quantify the degree of association between customer requirements and design features.Based on this symbolic weighting system, the functional requirement design matrix C_ij_ is constructed. This matrix forms the core “house” structure within the HOQ, visually and numerically expressing how effectively each technical feature addresses specific user needs.

In the fourth step, the right wall of the House of Quality (HOQ) is constructed. Based on the needs of chronic disease patients (represented by the left wall) and the functional requirement design matrix C_ij_ (the “room”), the relative importance of the needs of MCC D_i_, as well as their corresponding quality weights D_i_’, are calculated. These results constitute the right wall of the HOQ, as shown in Equations 6 and 7.


(6) $$D_{i}=\frac{\omega_{i}}{R_{i}}$$

(7) $$D{'}_{i}=\frac{D_{i}}{\sum_{i=1}^{m}D_{i}}$$

In the fifth step, the floor of the House of Quality (HOQ) is constructed. By integrating the previously established components of the HOQ, the functional importance of design requirements E_j_ and their corresponding relative importance values E_j_ ‘ are calculated, as shown in Equations 8 and 9.


(8) $$E_{j}=\sum_{i=1}^{m}D{'}_{i}C_{ij}$$

(9) $$E{'}_{j}=\frac{E_{j}}{\sum_{i=0}^{m}E_{j}}$$

**Figure 7:**
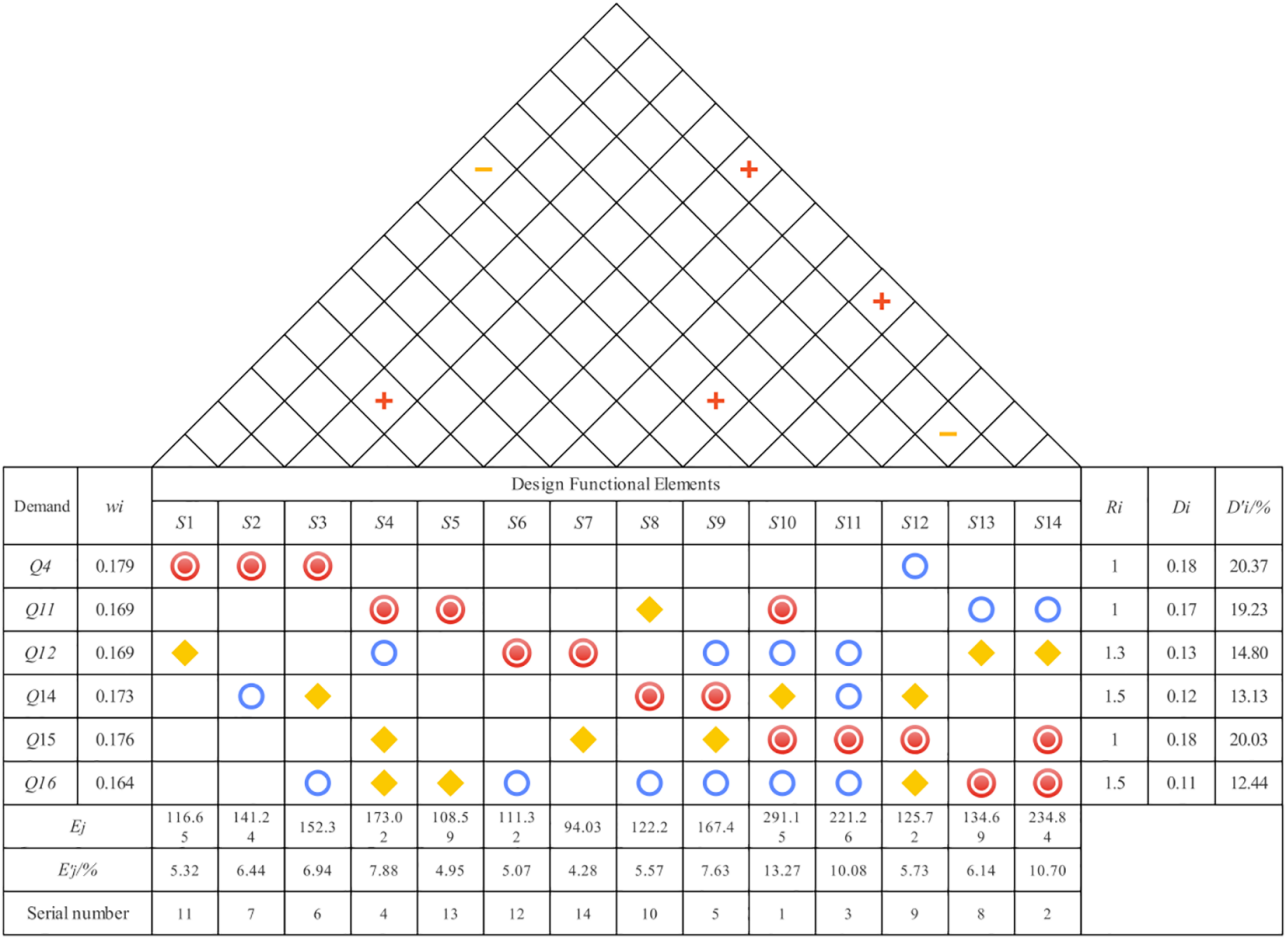
Design quality.

**Figure 8:**
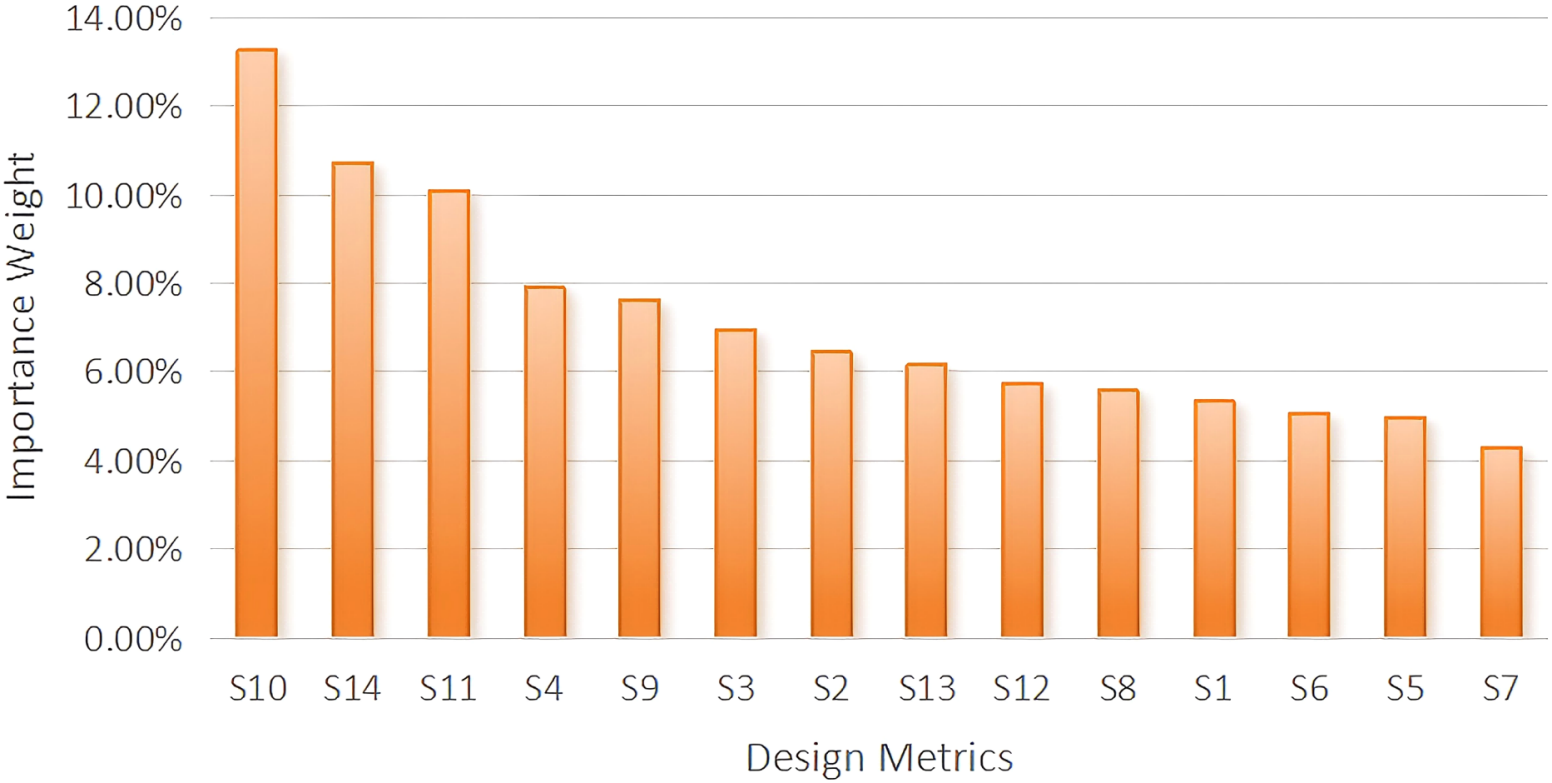
Functional design relative importance results.

Based on the preceding steps, the Quality House for the MCC patient museum experience design is constructed, as shown in **Figure 7**. From this, the prioritization of the relative importance of the functional elements is derived, which is presented in **Figure 8**.

### 5.4 Design strategy

**Figure 9:**
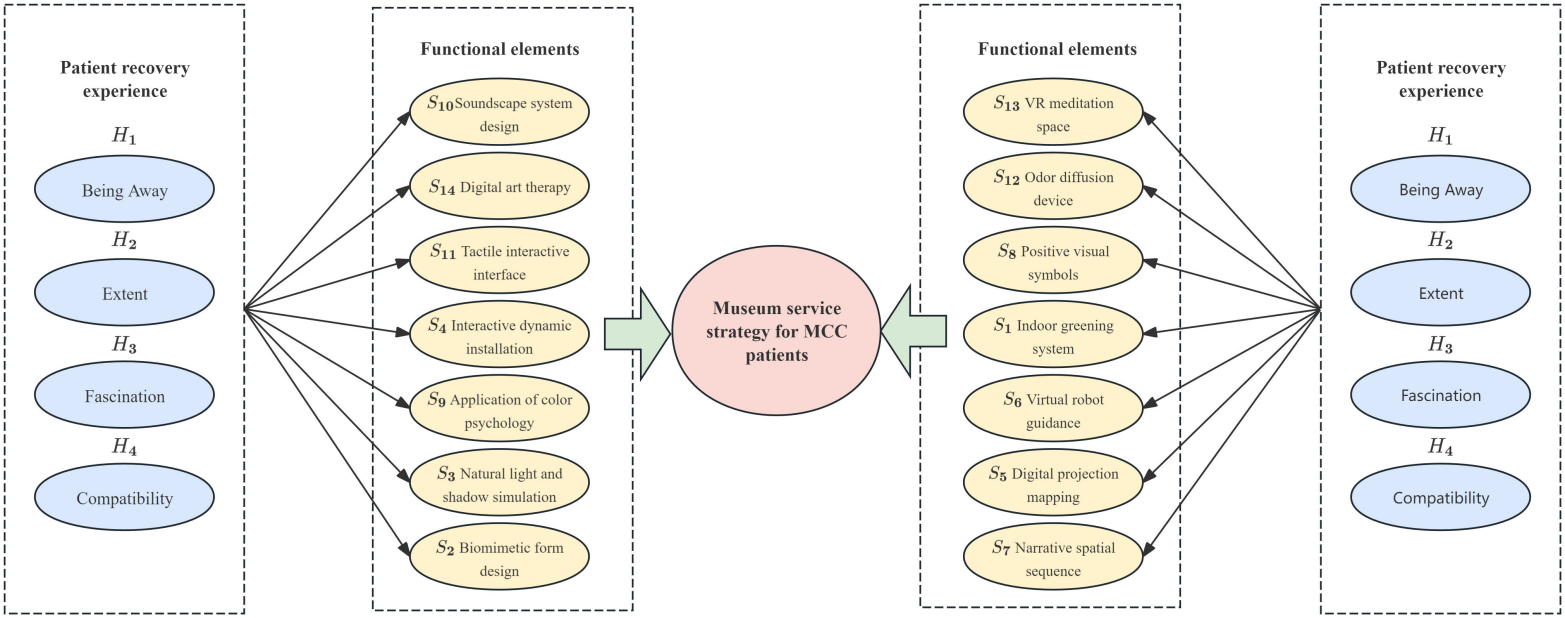
Design strategy model.

Drawing on systematic research in the theory of healing environments, this study analyzes the four characteristics: Being Away, Extent, Fascination, and Compatibility, and proposes an innovative strategy framework for museum service experience design tailored to MCC patients, as illustrated in **Figure 9**. In response to the lack of a systematic service design for MCC patients in the current museum sector, this study constructs a digitally-enabled immersive healing service design paradigm by integrating multidimensional demand analysis methods, including the Kano model, Quality Function Deployment (QFD), and the PUGH Decision Matrix, which broadens the scope of healing for MCC patients, generates greater therapeutic value, and aids in the development of a smart, intelligent museum service system for MCC patients.

#### 5.4.1 Escape design strategies

The escapist design seeks to offer MCC patients an opportunity to escape the stresses of daily life, enabling them to find inner peace and restoration within the museum environment. A core component of this design is the soundscape system. By incorporating background sound effects or natural sounds (e.g., running water, birdsong), the museum creates a tranquil space that aids patients in relaxing and escaping external stresses. Complementing digital art healing, interactive art installations can be designed to leverage the therapeutic power of artworks, alleviating patients’ anxiety and fostering emotional recovery through the interplay of vision and sound. To further enhance participation, museums should incorporate tactile interactive interfaces, enabling patients to experience the environment through touchscreens or textured exhibits, thereby diverting attention and alleviating anxiety related to health concerns. Additionally, interactive and dynamic devices can be developed to adjust display content based on patient interactions, thereby enhancing participation and supporting gradual relaxation during the experience. To enhance the escape experience, simulations of natural light and shadow can be designed to help patients feel closer to nature and relax further through the depiction of light and shadow changes across different times of day. Finally, the bionic design incorporates natural forms (e.g., those of plants and animals) to strengthen the patients’ connection to nature, providing additional comfort and emotional healing. Through these designs, the museum creates an immersive and soothing environment that enables MCC patients to escape daily stress and achieve emotional recovery.

#### 5.4.2 Extended design strategy

The extended design enables MCC patients to explore the museum freely by offering ample space and rich sensory experiences, thereby regulating mood and promoting recovery. Firstly, the design of VR meditation spaces immerses patients in natural, artistic, or historical scenes through virtual reality technology, aiding relaxation and alleviating emotional stress associated with chronic illness. When combined with digital projection mapping, museums can design dynamic projections of artworks or natural landscapes onto walls or floors, enhancing the spatial visual extensibility and enabling patients to perceive environmental changes and expansions during interaction, thus further promoting emotional relief. Narrative spatial sequences, meanwhile, integrate the narrative nature of the exhibition content by establishing distinct visiting areas and experiential paths, guiding patients to transition smoothly from one space to another, thus gradually relaxing and alleviating psychological pressure during the exploration process. Finally, the application of color psychology fosters a sense of comfort and tranquility for patients by leveraging the psychological effects of color. Warm tones such as soft orange and yellow evoke a sense of warmth, while cooler shades of blue and green help soothe emotions and alleviate anxiety. Through these design strategies, the museum creates an immersive, relaxing, and healing environment for patients.

#### 5.4.3 Captivating design strategies

Central to captivating design is the ability to distract patients from negative emotions by engaging their attention and stimulating their interest. First, positive visual symbols are crafted to convey uplifting and pleasant emotional messages through eye-catching art installations and signage. Bright colors, unique graphics, and forms can attract patients and motivate them to engage actively, thereby effectively reducing anxiety and depression and fostering a pleasant experience. Odor diffusion devices are also crucial elements in enhancing the environment’s attractiveness. Museums can install scent diffusion devices in designated areas to release pleasant odors, such as floral and woody scents, thereby enhancing the environment’s attractiveness through olfactory stimulation, while simultaneously regulating emotions through the therapeutic effects of scent, helping patients feel relaxed and comfortable. Additionally, the design of tactile interactive interfaces contributes to the fascination element. Through various tactile exhibits or devices, patients can touch and feel the texture and changes of the displays. This interactive approach captures their attention, enhances engagement, and helps divert attention from negative emotions, thereby reducing stress. The incorporation of virtual robot guidance also adds interest and interactivity. Virtual robots can offer friendly guidance to patients, assist in familiarizing them with the museum layout, provide real-time touring advice, and further enhance engagement and interest through interaction. These designs help distract patients from negative emotions and promote emotional regulation and recovery through multisensory stimulation and interaction.

#### 5.4.4 Compatibility design strategy

The purpose of compatibility design is to ensure the museum environment aligns with the needs of MCC patients, offering a space that is comfortable, flexible, and easy to adapt to. First, the indoor greening system is a key element of compatibility design. By incorporating plants into the museum and creating a natural, comfortable environment, this system not only beautifies the space but also alleviates emotional stress and enhances overall comfort through the greenery and natural atmosphere. Additionally, personalized settings and adaptive design are crucial. The museum can offer personalized settings, such as adjustments to volume, light intensity, and air humidity, allowing patients to modify environmental parameters based on their health conditions and needs, thereby preventing over-stimulation or discomfort and ensuring a comfortable experience. Simultaneously, accessible design and navigation systems are crucial, particularly for patients with physical or cognitive disabilities. Museums should design accessible routes, rest areas, wheelchair access, and other facilities to ensure that all patients can visit comfortably and smoothly. Additionally, simplified guiding systems, such as audio guides and touchscreen guides, will assist patients in understanding exhibit content and spatial layout, allowing them to easily adapt to the museum environment. Through these design strategies, the museum creates a comfortable, flexible, and adaptable environment for MCC patients, facilitating an enjoyable visiting experience while promoting emotional regulation and physical and mental recovery.

### 5.5 Comprehensive evaluation of museum design proposals based on the pugh matrix

1. Initial screening of design options

**Figure 10:**
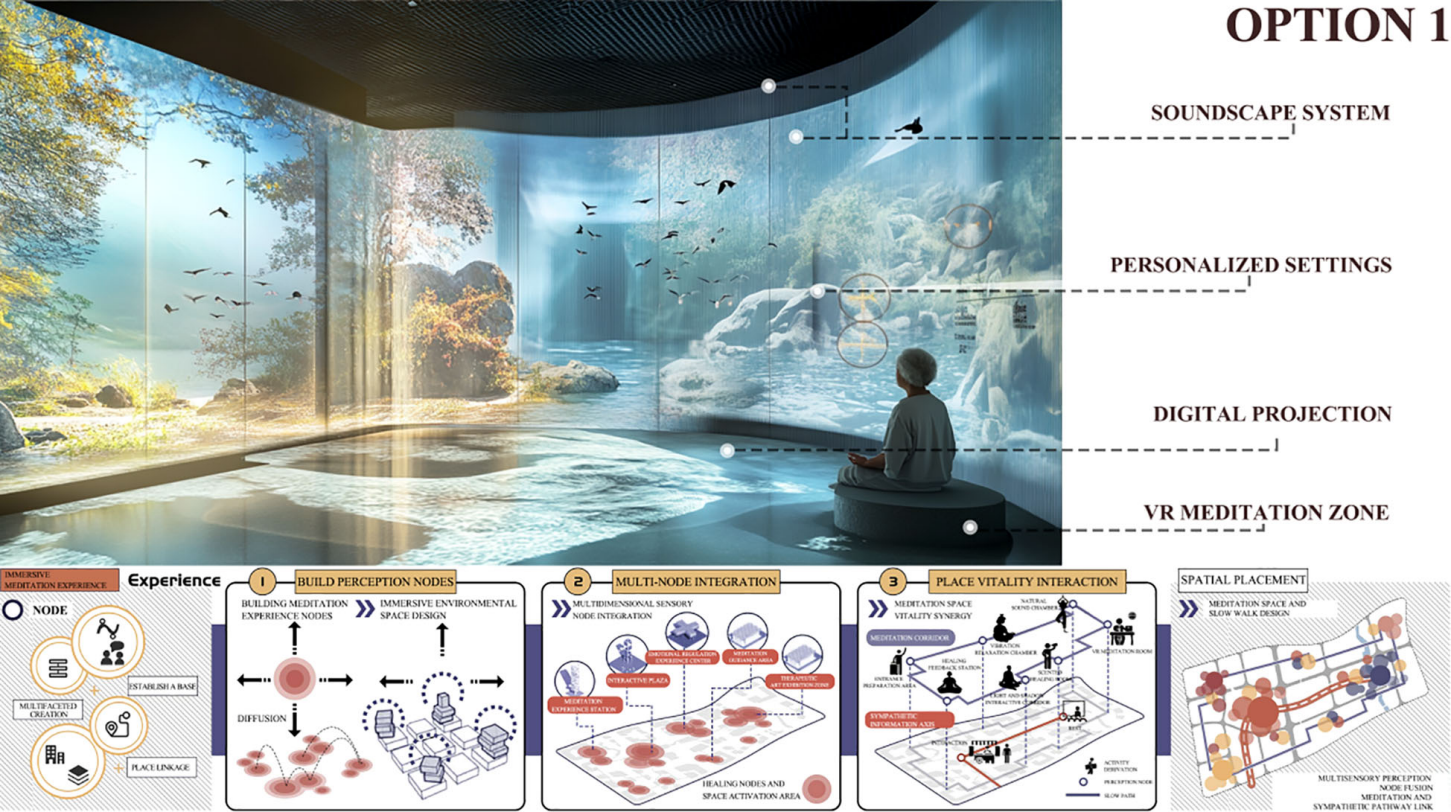
Programme I.

Based on the QFD Quality House analysis of the importance of functional requirements, four distinct conceptual museum design solutions were developed after two design iterations.They all encompass the top-ranked key functional requirements and reflect different design concepts and implementation approaches. At the same time, they exhibit distinct variations in spatial layout, interaction methods, and presentation media, thereby avoiding homogenization. Moreover, expert evaluations confirmed that each scheme possesses fundamental technical feasibility and implementation sustainability, making them suitable as candidate solutions for subsequent comparison and optimization.featuring the following specific design elements:

Programme 1: This program creates an immersive meditation environment for emotional regulation and psychological recovery through multidimensional sensory intervention and technological empowerment. The core of the space is the natural soundscape system, which dynamically plays 360° surround ecological sound sources (such as birdsong and flowing streams), integrated with intelligent noise reduction walls to isolate external disturbances. The virtual reality meditation pod offers natural scenes such as the deep sea and forests, supports eye tracking and brainwave feedback, and optimizes scene complexity in real-time to enhance concentration. The interactive light and shadow device diverts attention and fosters positive emotions through 4D projection and gesture recognition technology, while the bio-adaptive system automatically adjusts the color temperature of the light and fragrance concentration based on heart rate, breathing, and other data to achieve personalized adaptation. Patients can follow a structured pathway while exploring freely, with accessibility incorporated into every aspect of the design. Long-term intervention can significantly alleviate depression symptoms in MCC patients and enhance emotional regulation, forming a closed-loop system of ‘natural healing - technology empowerment - data optimization’ that balances professionalism and inclusiveness, as shown in **Figure 10**.

**Figure 11:**
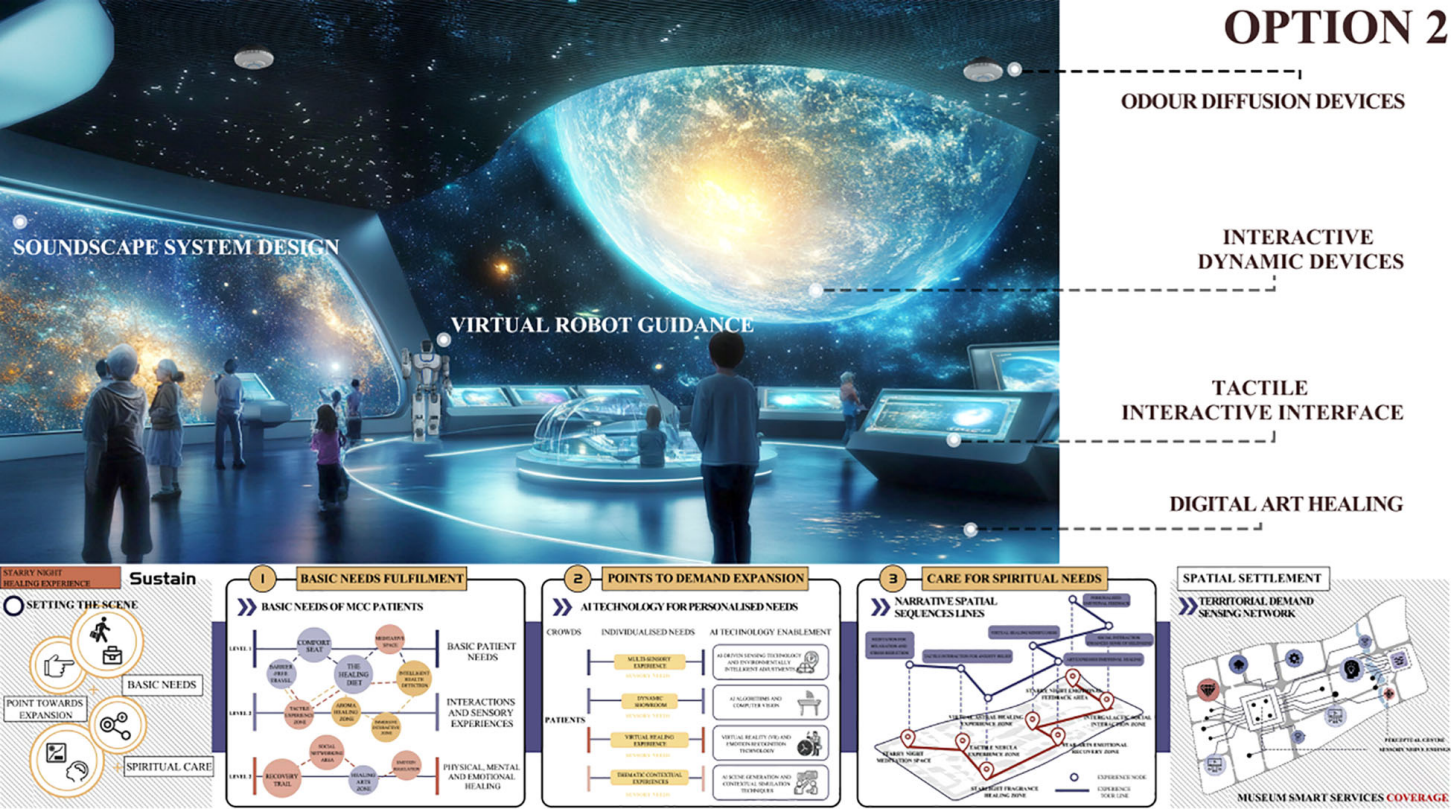
Programme II.

Programme 2: The Star Healing Experience program encourages MCC patients to explore the museum freely through enhanced sensory stimulation and interactive design, thus promoting emotional recovery and physical rehabilitation. A narrative spatial sequence within the museum integrates various exhibits to help patients gradually relax and ease their emotions throughout their visit. Within the museum, patients engage with exhibits through tactile interactive interfaces. The touchscreen and various textured exhibits assist in diverting attention, reducing anxiety, and providing a pleasurable experience. Interactive dynamic devices, in conjunction with spatial dynamics (e.g., sensory light walls, motion-responsive sculptures, and sound-triggered interactive platforms), change in real time based on audience behavior, enhancing engagement and autonomy. These devices promote physical participation and sensory mobilization, stimulating the desire for exploration and self-regulation. Additionally, digital projection mapping technology displays dynamic artworks that enhance spatial extensibility, enabling patients to discover new visual and tactile experiences during exploration, thus further stimulating interest and participation. Scent diffusion devices are also installed throughout the museum, using fragrance to regulate ambiance, soothe patients’ emotions, and enhance the environment’s attractiveness. Furthermore, the museum is designed with barrier-free facilities and an adaptive guiding system to ensure accessibility for all patients, including those with mobility impairments. It features a personalized spatial layout and clear guidelines to help patients feel comfortable and enjoy their visit. The overall program aims to create an interactive and healing museum environment, as shown in **Figure 11**.

**Figure 12:**
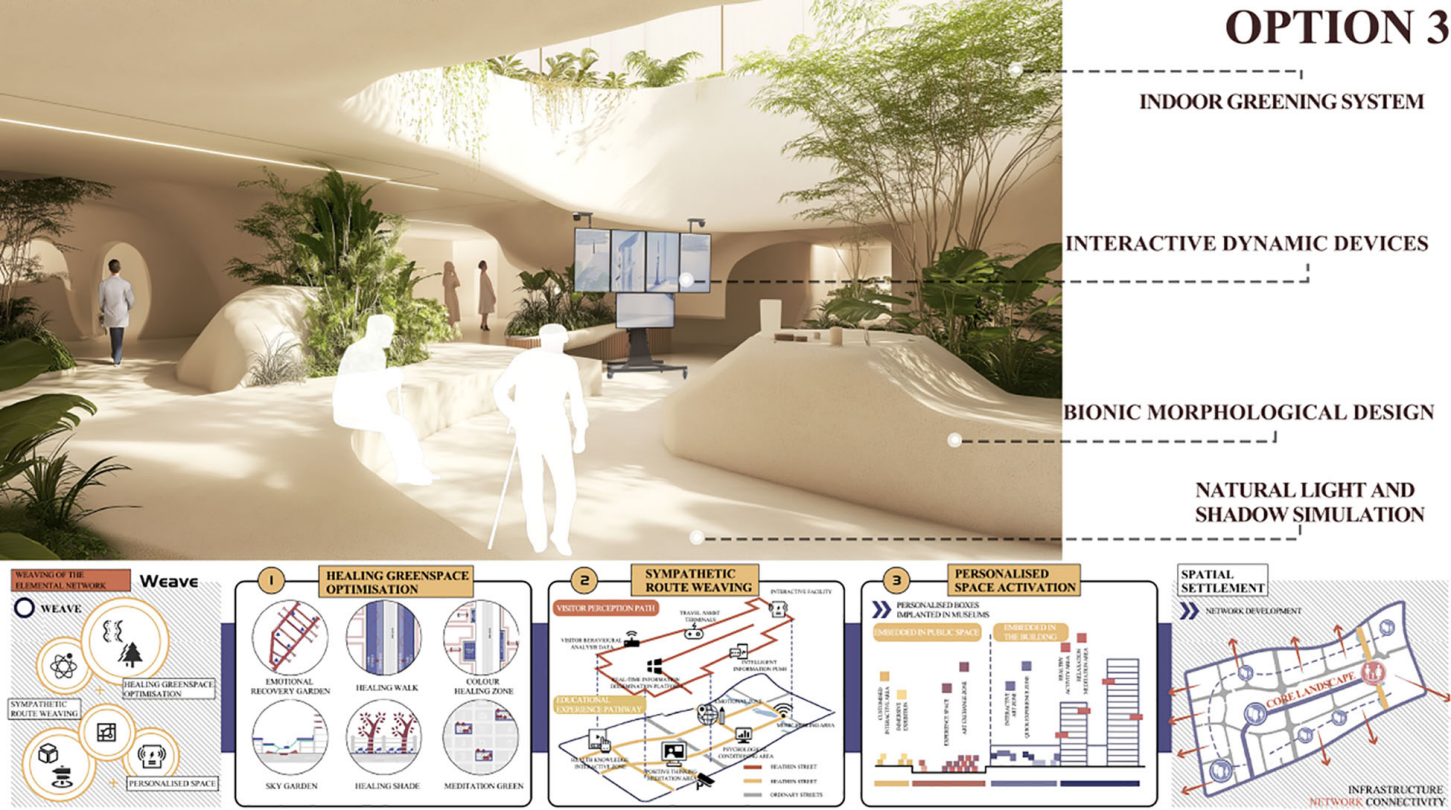
Programme III.

Programme 3: The Emotional Recovery and Education Program aims to assist MCC patients in healing and improving their health perceptions within the museum by integrating emotional regulation with educational experiences. The museum environment incorporates bionic forms, such as simulated plants and natural elements, alongside soothing background sound effects to create a peaceful and healing space, enabling patients to temporarily disconnect from daily stresses and feel relaxed. Positive visual symbols and interactive installations guide patients to actively engage with the environment, with art installations that captivate their interest while effectively diverting attention from negative emotions. Additionally, personalized spaces and a simplified navigation system ensure that each patient can adapt the environment to their individual needs, helping them better understand the exhibits while ensuring a comfortable and smooth visit. The overall design aims to provide patients with a museum experience that serves the dual purposes of emotional regulation and education, as shown in **Figure 12**.

**Figure 13:**
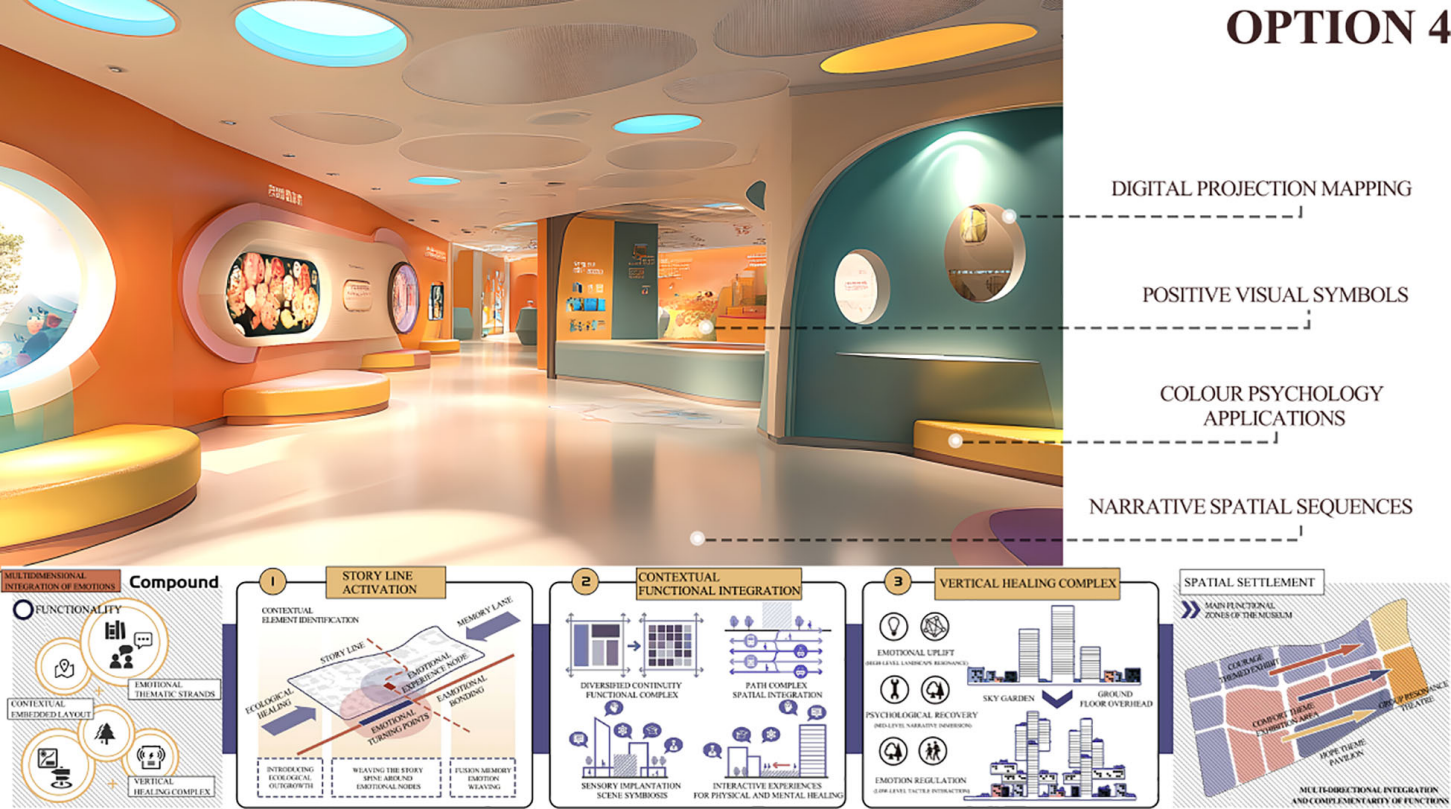
Programme IV.

Option 4: Emotionally Driven Interactive Storytelling Experience. Through emotionally themed interactive storylines, MCC patients are guided through narrative situations in the museum to facilitate emotional regulation and psychological recovery. The museum features several emotion-focused exhibits (e.g., Hope, Courage, Comfort) that allow patients to engage with emotionally charged narrative situations during their visit. Through interactive screens, augmented reality (AR) technology, and projection mapping, patients can not only observe the presentation of the stories but also influence their development, enhancing the sense of participation. The museum, as a vertical healing complex, is designed with a three-tiered structure: emotional regulation (bottom-tier tactile interaction), psychological recovery (middle-tier narrative immersion), and emotional sublimation (top-tier landscape resonance). The adaptive navigation system and accessibility facilities ensure that each patient can tailor the visit experience to their individual health needs, making the process both therapeutic and capable of stimulating emotional resonance, further promoting psychological recovery, as shown in **Figure 13**.

2. Comprehensive evaluation of design options

We formed an evaluation panel to conduct an initial assessment of four museum experience design solutions tailored for MCC patients, with the aim of ensuring a professional and in-depth functional evaluation of the design proposals. The panel consisted of 35 MCC patients, 10 patient caregivers, 5 interaction designers, 7 curatorial designers, and 3 academic experts. All members were selected based on their professional background and relevant experience. Prior to inclusion, all MCC patients underwent cognitive function and mental health screenings to ensure their ability to effectively participate in the evaluation and provide meaningful feedback. Interaction designers and curatorial designers were selected based on their professional qualifications and practical experience in museum design, accessible design, and patient experience. Academic experts were chosen for their academic achievements and research expertise in museum design, gerontology, and patient experience studies. The composition of this evaluation panel ensured the comprehensiveness, professionalism, and scientific rigor of the evaluation process.

First, the design team identified Scheme 1 as the baseline scheme for evaluation, based on the design function elements and weightings analyzed in the QFD, with all grades initially set to 3. Second, the four conceptual design schemes were assessed using the expert panel scoring method, with scores assigned on a scale of 1-5 (where 1 indicates very poor, 2 indicates slightly poorer, 3 indicates the same, 4 indicates marginally better, and 5 indicates better). Lastly, the evaluation of the four design schemes was conducted for each design function element grade from S1 to S14, according to the evaluation criteria outlined in the project assessment guidelines and benchmarked against Programme 1 for comparison. The scores, weighted by the importance of the design criteria, were used to calculate the comprehensive score for each design program. The results are shown in **Table 15**, and the calculation formula is as follows according to Equations 10–14:

**Table 15:** Museum experience design composite score.

Indicator	Weights	Programme 1		Programme 2		Programme 3		Programme 4	
		Rating	Score	Rating	Score	Rating	Score	Rating	Score
*S*1	5.32%	3	0.16	3	0.16	3	0.16	2	0.11
*S*2	6.44%	3	0.19	3	0.19	4	0.26	2	0.13
*S*3	6.94%	3	0.21	3	0.21	4	0.28	2	0.14
*S*4	7.88%	3	0.24	4	0.32	4	0.32	3	0.24
*S*5	4.95%	3	0.15	4	0.20	2	0.10	4	0.20
*S*6	5.07%	3	0.15	4	0.20	2	0.10	2	0.10
*S*7	4.28%	3	0.13	3	0.13	2	0.09	4	0.17
*S*8	5.57%	3	0.17	3	0.17	4	0.22	4	0.22
*S*9	7.63%	3	0.23	3	0.23	2	0.15	4	0.31
*S*10	13.27%	3	0.40	4	0.53	2	0.27	4	0.53
*S*11	10.08%	3	0.30	4	0.40	2	0.20	3	0.30
*S*12	5.73%	3	0.17	4	0.23	3	0.17	2	0.11
*S*13	6.14%	3	0.18	3	0.18	3	0.18	3	0.18
*S*14	10.70%	3	0.32	4	0.43	2	0.21	3	0.32
Overall programme rating		3.00		3.58		2.71		3.06	
Standard Deviation		0.077		0.119		0.072		0.117	
Variance		0.006		0.014		0.005		0.014	
Interquartile Range		0.098		0.162		0.125		0.17

1. Indicator score for the jth design function element for the kth programme.

(10) $$F_{jk}=E{'}_{j}\cdot d_{jk}$$

2. Calculation of programme composite scores.

(11) $$F_{k}=\sum_{j}F_{jk}$$

3. Standard Deviation

(12) $$SD=\sqrt{\frac{\sum_{i=1}^{n}{\left(x_{i}-\bar{x}\right)}^{2}}{n-1}}$$

4. Variance

(13) $$Var=\frac{\sum_{i=1}^{n}{\left(x_{1}-\bar{x}\right)}^{2}}{n-1}$$

5. Interquartile Range

(14) $$\text{IQR}={\text{Q}}_{3}-{\text{Q}}_{1}$$

3. Satisfaction Assessment

An adapted version of the UEQ-S scale tailored to MCC healing environments was employed to assess museum experience satisfaction (67).The scale covered four dimensions: emotional involvement, perceived usefulness, sensory comfort, and satisfaction. It was rated on a 7-point Likert scale and administered both before and after the intervention.

The results indicated that post-intervention satisfaction in the experimental group was significantly higher than in the control group (5.87 ± 0.85 vs. 4.52 ± 0.95), with a statistically significant difference between groups (t(58) = 5.79, p< 0.001). The relative improvement was 29.87%, suggesting that the proposed design effectively enhanced the satisfaction experience of MCC patients.

4. Museum Experience Design Final Design Plan

According to the comprehensive evaluation results presented in **Table 15**, Scheme 2 achieved the highest overall score (3.58), clearly outperforming the other three schemes. This finding indicates that its overall design effectiveness was most highly recognized by both users and experts.

Although the standard deviation (0.119) and interquartile range (0.162) of Scheme 2 were slightly higher than those of the other schemes, this variation primarily stemmed from its outstanding performance on several high-weight indicators (e.g., S4, S10, S11, and S14), which substantially elevated the overall mean score. This distribution pattern reflects Scheme 2’s exceptional performance across key design dimensions rather than instability in the data.

Considering both the average performance and the importance of indicator weights, Scheme 2 demonstrated the best results in terms of “performance improvement” and “user satisfaction,” and can thus be identified as the optimal design solution in this study.

**Figure 14:**
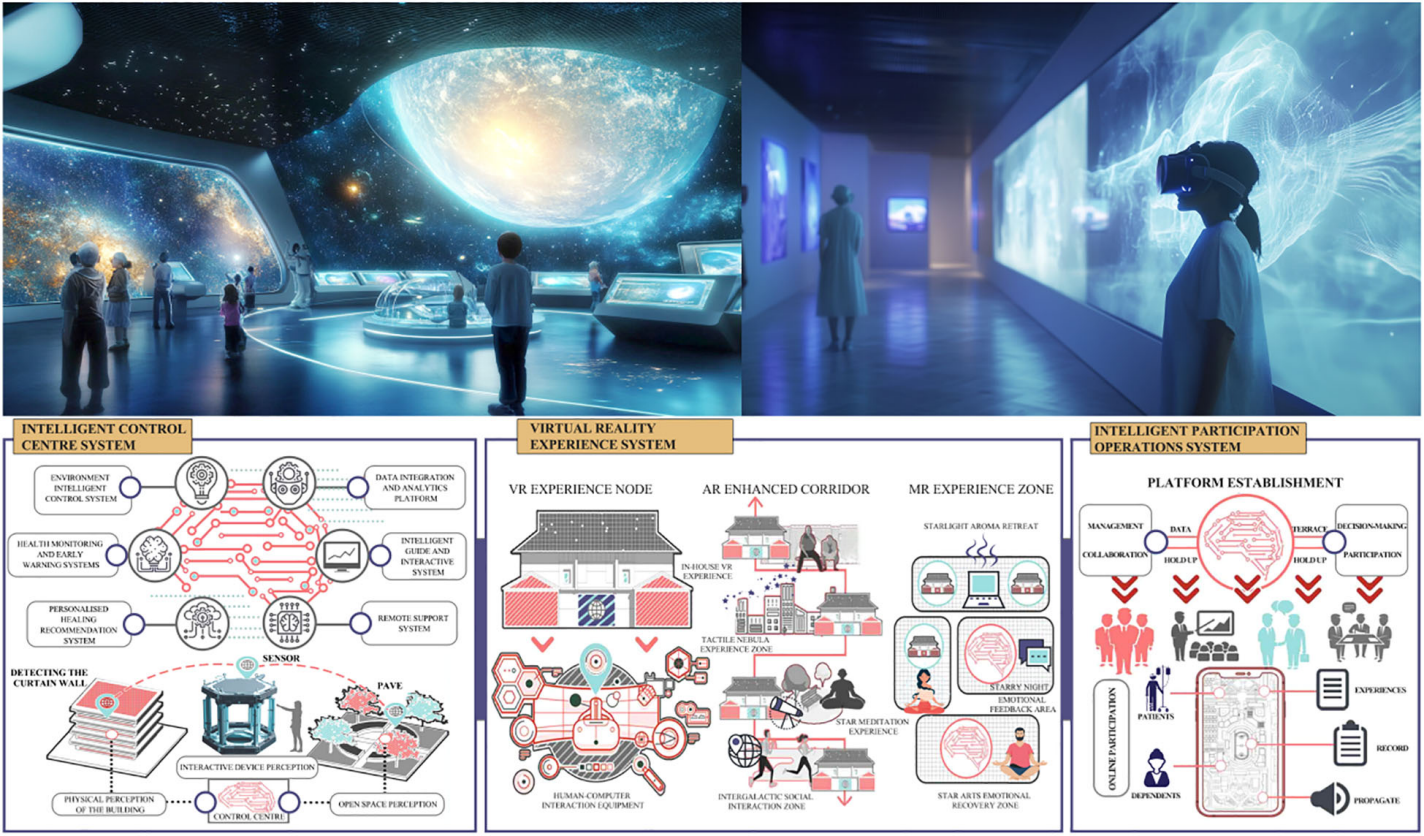
Star healing experience programme.

Centered on multi-sensory stimulation and autonomous exploration, this solution effectively addresses the key needs of MCC patients for low cognitive load and high comfort interventions during emotional recovery. Through a carefully arranged narrative spatial sequence, the Starry Sky Healing Experience integrates visual, tactile, and olfactory interactions, complemented by dynamic digital projection, soundscape system design, richly textured tactile interfaces, and a natural fragrance diffusion system. This creates a highly immersive, continuous, and gradual emotional healing pathway, significantly reducing psychological resistance and cognitive load during the healing process. Compared to Programme 1, which emphasizes high-tech immersive intervention, Programme 3, which focuses on educational cognitive enhancement, and Programme 4, which relies on interactive emotional narratives, the Starry Sky Healing Experience prioritizes sensory instincts. It alleviates anxiety and reconstructs positive emotions through natural guidance, without overburdening patients’ cognitive resources, making it especially suitable for patients with chronic illnesses and cognitive limitations. Additionally, the program emphasizes low-threshold, highly inclusive spatial accessibility design, featuring comprehensive barrier-free facilities and an intelligent adaptive navigation system. This enables patients with diverse physical and cognitive conditions to complete the exploration experience independently and safely, further enhancing their self-efficacy and subjective initiative, as shown in **Figure 14**.To minimize the risk of discomfort or anxiety, artworks conveying strong negative emotions were curated separately from those with more therapeutic qualities, thereby providing a gradually guided experience and preventing audiences from being exposed to excessive emotional impact without appropriate transition. In addition, background information about the artworks was provided through textual descriptions, audiovisual materials, and other media to facilitate a deeper understanding of the creative intent and historical context, thereby reducing potential misunderstandings or feelings of discomfort.

Immersive Escape: The Starry Night Meditation Experience program fully realizes the “Being Away” feature of the healing environment theory by creating a highly heterogeneous immersive sensory environment. The design incorporates the Starry Night Meditation Space, Tactile Nebula Experience Zone, Starlight Fragrance Healing Zone, Star Arts Emotional Recovery Zone, and other spaces to create an immersive environment distinct from the patient’s daily surroundings. The incorporation of these spaces forms an immersive environment that fosters psychological “remoteness” for patients upon entering the museum. This detachment from stress creates an independent, focused space conducive to emotional recovery. This intervention, based on both physical and psychological “escape,” effectively mitigates the patient’s initial anxiety, laying a solid foundation for the subsequent reconstruction of positive emotions.

Coherent Exploratory Experience: The Starry Sky Healing Experience program creates a holistic and unified environment with deep exploratory possibilities through fluid spatial sequences and multi-sensory gradual transitions. The natural gradation of colors, materials, aromas, and sound effects between exhibition areas diminishes spatial boundaries and strengthens the continuous perceptual experience, enabling patients to explore the museum uninterrupted while maintaining focus and a state of “flow.” Simultaneously, the moderate enrichment of environmental elements at the multidimensional sensory level prevents the experience from becoming monotonous and enhances the durability and breadth of emotional intervention.

Natural Sensory Attraction: Fascination is fully realized in the Astral Healing Experience. Through dynamically changing star projections, tactile interactive interfaces, and synchronized scent diffusers, the environment continuously attracts the patient’s attention involuntarily, preventing cognitive overload. Patients unconsciously focus on the environment, reducing excessive attention and rumination on negative emotions, thereby facilitating cognitive recovery and relieving psychological pressure. Subtle changes in environmental elements and multi-sensory integration ensure sustained attraction, playing a crucial role in guiding emotional transformation.

Highly Adaptable Spatial Design: The Star Healing Experience is grounded in a highly humanistic design concept, ensuring strong compatibility between the environmental features and the physiological and psychological states of the patients. Inside the space, barrier-free facilities are extensively integrated, including low booths, ramps, and auxiliary guide signs, along with an intelligent adaptive guide system that automatically optimizes the path and pace of the visit based on the patient’s movement trajectory and areas of interest. Patients can customize the exploration process according to their personal health status and preferences, thereby avoiding conflicts between environmental requirements and their abilities. This customization significantly enhances the affinity of the healing environment and the effectiveness of the intervention.

## 6 Discussion

This study, based on the KANO-QFD-PUGH method, provides a structured and systematic approach for requirement elicitation and solution optimization in museum design for MCC patients, particularly offering new development processes and ideas in aligning user and designer needs. Previous studies have shown that the Kano model, QFD methodology, and Pugh Matrix have demonstrated advantages in optimizing design solutions and capturing user requirements across various fields (68). This study further empirically validates that the method effectively enhances patient satisfaction in museum therapeutic design, thus confirming its applicability and value in this field.

Furthermore, this study focuses on comprehensive historical and art museums. Although the findings are applicable in this domain, the applicability and feasibility in other types of museums (such as science museums, children’s museums, etc.) require further investigation. Different types of museums have distinct functions and target audiences, and their therapeutic effects and experiential value may vary. Existing literature indicates that different museum types can have varying impacts on visitors’ emotional experiences and cognitive load. Therefore, future research should expand to include different types of museums to assess the generalizability and applicability of the conclusions drawn from this study.

This study presents several limitations regarding the therapeutic solutions proposed in museum design. Firstly, the duration and frequency of museum visits may impact the sustainability and clinical significance of the therapeutic effects. The current study did not sufficiently explore these variables; therefore, future research should consider the long-term effects of visit frequency, duration, and the patient’s health condition on therapeutic outcomes. Secondly, although this study explored the potential of museum therapy, it has not conducted a direct comparison with other non-pharmacological interventions, such as psychotherapy or meditation. The relative advantages of these interventions in terms of therapeutic effects still require further validation.Museum therapy should not be regarded as a substitute for standard clinical treatment but rather as a complementary, community-based intervention that enhances conventional care by promoting emotional well-being and social engagement.

Finally, museums face practical limitations, such as crowd control, resource allocation, and artifact preservation (e.g., lighting, humidity control). The introduction of elements such as plants, scents, or sounds may conflict with the preservation requirements of artifacts. Therefore, future research should balance the therapeutic potential with these practical challenges, ensuring that the design optimizes the therapeutic environment without compromising artifact preservation. Additionally, the KANO-QFD-PUGH method still faces challenges in practical applications, such as dynamic demand modeling, the identification of latent needs, and long-term adaptability assessments. Future studies could incorporate lifecycle experience management and multimodal data analysis methods to enhance the scientific rigor and precision of the design.

## 7 Conclusion

This study theoretically explores the issues of museum design and development aimed at optimizing the experience of MCC patients, introducing the Kano-QFD-PUGH integrated product development process (69). This approach overcomes the limitations of traditional museum design, which often heavily relies on the designer’s subjective judgment. The method systematically extracts user requirements, helps the design team identify development priorities, and partially optimizes the design selection process. Through the application of practical case studies, this research demonstrates the potential value of this method in optimizing the experience of MCC patients. However, as an exploratory study, the conclusions lack further validation through large-scale quantitative data. Therefore, the contribution of this study lies mainly in providing a reference design approach and process demonstration, which needs to be further tested for applicability with a larger sample size and empirical research in the future.

## Data Availability

The original contributions presented in the study are included in the article/**Supplementary Material**. Further inquiries can be directed to the corresponding author.
